# Total Synthesis and Antimicrobial Evaluation of 23-Demethyleushearilide and Extensive Antimicrobial Evaluation of All Synthetic Stereoisomers of (16*Z*,20*E*)-Eushearilide and (16*E*,20*E*)-Eushearilide

**DOI:** 10.3390/molecules24193437

**Published:** 2019-09-22

**Authors:** Takayuki Tonoi, Takehiko Inohana, Teruyuki Sato, Yuuki Noda, Miyuki Ikeda, Miku Akutsu, Takatsugu Murata, Yutaro Maekawa, Anna Tanaka, Rio Seki, Misako Ohkusu, Katsuhiko Kamei, Naruhiko Ishiwada, Isamu Shiina

**Affiliations:** 1 Department of Applied Chemistry, Faculty of Science, Tokyo University of Science, 1-3 Kagurazaka, Shinjuku-ku, Tokyo 162-8601, Japan; inohanat@nissanchem.co.jp (T.I.); sato-teruyuki@gms.kanto.co.jp (T.S.); 1318598@ed.tus.ac.jp (Y.N.); 1318508@ed.tus.ac.jp (M.I.); 1319503@ed.tus.ac.jp (M.A.); A29215@rs.tus.ac.jp (T.M.); 1318617@ed.tus.ac.jp (Y.M.); at.schokolade@gmail.com (A.T.); 2 Department of Infectious Diseases, Medical Mycology Research Center, Chiba University, 1-8-1 Inohana, Chuo-ku, Chiba-shi, Chiba 260-8673, Japan; rseki@chiba-u.jp (R.S.); misako@office.chiba-u.jp (M.O.)

**Keywords:** eushearilide, demethyl congener, total synthesis, lactonization, MNBA, antimicrobial activity

## Abstract

A novel stereoisomer of eushearilide, 23-demethyleushearilide, was synthesized, and the structure–activity relationships of this compound along with known eushearilide stereoisomers were investigated in order to design novel lead compounds for the treatment of fungal infections. It was discovered that all of these congeners, together with the natural product, exhibited a wide range of antimicrobial activity against not only fungi but also against bacteria, including methicillin-resistant *Staphylococcus aureus* (MRSA) and vancomycin-resistant enterococci (VRE).

## 1. Introduction

The occurrence of life-threatening mycoses is currently increasing in immunocompromised hosts, including cancer chemotherapy patients, patients infected with human immunodeficiency virus (HIV), and people receiving organ transplant-associated immunosuppressive therapy [1]. Such fungal infections are treated using three principal types of antifungal agents—polyenes, azoles, and candins—which are developed and employed in widespread clinical uses [2]. Among these, amphotericin B, a typical polyene macrolide antibiotic [3], has an especially broad antifungal spectrum against pathogenic fungi such as *Aspergillus* spp., *Candida* spp., and *Cryptococcus* spp. Amphotericin B is therefore utilized to treat systemic mycoses, including clinically important antifungals, although it has toxicity issues and may cause severe side effects, including renal failure. Furthermore, it was recently reported that *Aspergillus fumigatus* has developed resistance to azoles [4]. Strains of pathogenic fungi with antifungal resistance have already spread widely throughout the world. However, since the number of clinically available antifungal drugs is quite limited, there is a continually increasing demand for more effective antifungal drugs to overcome these intractable mycoses.

**Figure 1:**
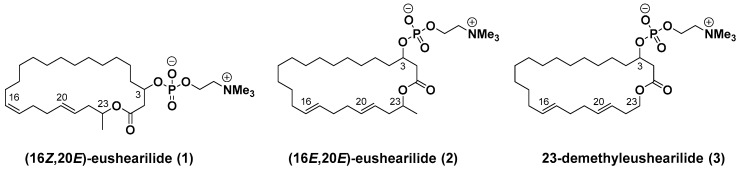
Structures of eushearilide congeners and the 23-demethyl derivative.

Eushearilide was first isolated from a culture of the fungus *Eupenicillium shearii* in 2006 during the course of screening antifungal compounds from fungal sources and was stereochemically and biologically characterized by Hosoe et al. [5]. The originally proposed structure, (16*Z*,20*E*)-eushearilide (**1**) (Figure 1), was shown to possess a 24-membered macrolactone framework as the main backbone with two stereogenic centers at the C3 and C23 carbons. Interestingly, this compound contains none of the 1,3-polyol moiety, conjugated polyene chain, and amino sugar moiety that are often present in polyene macrolide antibiotics, such as amphotericin B, natamycin, and nystatin. Instead, it was reported that eushearilide has nonconjugated dienes at the C16 and C20 carbons and a phosphorylcholine group at the C3 carbon. The relative and absolute configurations of the two stereogenic centers (C3 and C23) were not determined at that time. Hosoe et al. also reported that eushearilide exhibited antifungal activity against various fungi and yeasts, including the human pathogens *Aspergillus* spp., *Trichophyton* spp., and *Candida* spp. However, the antibacterial activity of eushearilide has not been fully investigated, although traces of antibacterial activity have been reported in the literature.

**Scheme 1:**
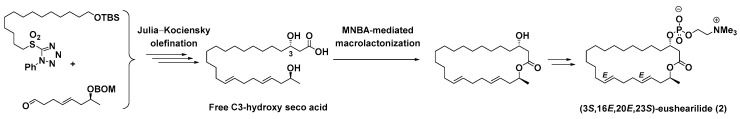
Synthetic outline for the preparation of naturally occurring eushearilide (**2**).

Our previous work has concerned the unique structural characteristics and promising structure–activity relationship of eushearilide and the total synthesis of this novel antibiotic. In 2015, the total syntheses of **1** and its diastereomers, (3*R*,16*Z*,20*E*,23*R*)- and (3*S*,16*Z*,20*E*,23*R*)-**1**, were achieved [6,7]. From the results of detailed NMR analyses of these compounds, it was found that natural eushearilide comprises a (16*E*,20*E*) configuration (as shown in eushearilide (**2**)) and not a (16*Z*,20*E*) configuration of the nonconjugated diene moiety at the C16 and C20 carbons. Thus, we addressed and first accomplished the total synthesis of naturally occurring eushearilide, (3*S*,16*E*,20*E*,23*S*)-(+)-**2** [8]. Moreover, in order to improve the yield and reduce the number of steps in the synthetic process, we thoroughly revised our previous method of synthesis, as shown in Scheme 1 [9], enabling us to obtain a sufficient amount of eushearilide derivatives for structure–activity relationship (SAR) studies. 

On the basis of synthetic methodology, a novel eushearilide derivative, 23-demethyleushearilide (**3**), was synthesized to investigate the effects on antimicrobial activity of the methyl substituent at the C23 carbon of eushearilide. We report herein the synthesis of compound **3** and the preparation of known structural congeners of eushearilide [9], whose antimicrobial activity against a wide spectrum of fungi and bacteria was also evaluated in detail.

## 2. Results and Discussion

### 2.1. Synthetic Strategy

**Scheme 2:**
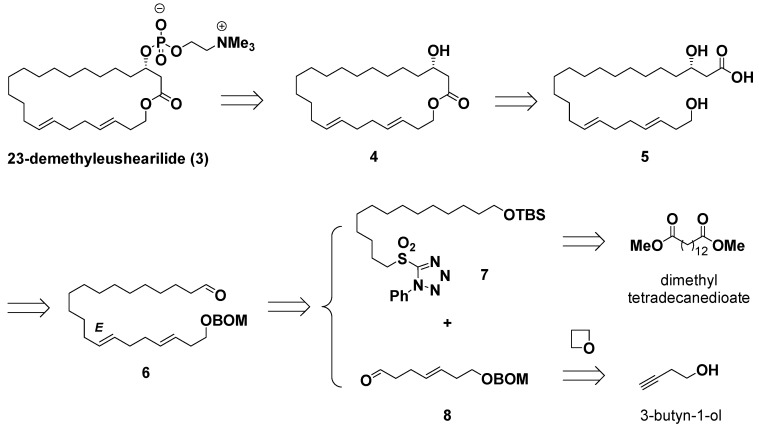
Retrosynthesis of 23-demethyleushearilide (**3**).

Our synthetic plan for compound **3** is shown in Scheme 2. The highly polar phosphorylcholine moiety [10] at the C3 position was introduced in the final stage. The macrolactonization of the seco acid **5** bearing a primary hydroxy group without a methyl substituent at the C23 carbon proceeded smoothly using 2-methyl-6-nitrobenzoic anhydride (MNBA)-mediated macrolactonization [11,12,13,14,15] in the presence of a nucleophilic catalyst. A β-hydroxy carboxylic acid moiety of the ring-closure precursor **5** could be synthesized by the asymmetric Mukaiyama aldol reaction [16,17,18] of aldehyde **6** with enol silyl ether. The (*E*)-alkene moiety at the C14 position of aldehyde **6** could be synthesized using the Julia–Kocienski olefination [19,20,21] between the known sulfone **7** and aldehyde **8** [9], both of which may be easily prepared through conventional transformations from commercially available products.

### 2.2. Synthesis of 23-Demethyleushearilide (**3**)

**Scheme 3:**
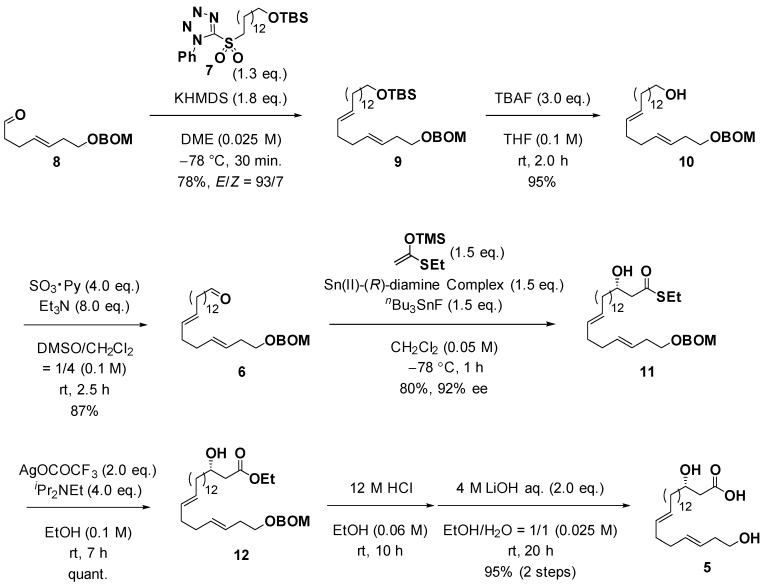
Synthesis of the free C3 hydroxy seco acid **5**.

On the basis of our previously established synthesis, the synthesis of the desired seco acid **5** was undertaken from the known aldehyde **8**, shown in Scheme 3. The Julia–Kocienski olefination of **8** with sulfone **7** in the presence of potassium hexamethyldisilazide (KHMDS) at −78 °C was performed to yield the desired diene **9** with high stereoselectivity (*E*/*Z* = 93/7). Deprotection of a primary *tert*-butyldimethylsilyl (TBS) ether in **9** and oxidation of the resulting hydroxy group provided the corresponding aldehyde **6**. The asymmetric Mukaiyama aldol reaction of **6** with an enol silyl ether derived from *S*-ethyl ethanethioate in the presence of an (*R*)-diamine-Sn(II) complex with *^n^*Bu_3_Sn(IV)F afforded the aldol product **11**, a good yield with high enantioselectivity (80%, 92% ee). After the transesterification of thioester **11** with silver trifluoroacetate in ethanol, ester **12** was obtained and was subjected to deprotection of the benzyloxymethyl (BOM) group followed by cleavage of the ethyl ester moiety to yield the free C3 hydroxy seco acid **5**.

**Scheme 4:**
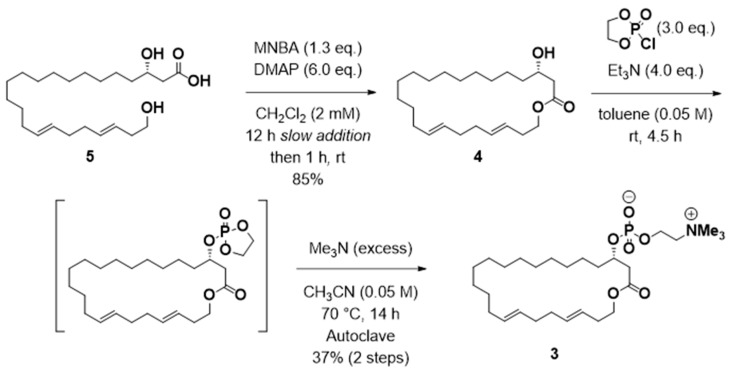
Macrolactonization followed by introduction of the phosphorylcholine moiety for the total synthesis of **3**.

Having successfully installed the desired stereogenic center at the C3 carbon in **5**, it was then necessary to form the macrocyclic ring via MNBA-mediated macrolactonization. The macrolactonization of **5**, which possessed a primary hydroxy group without a methyl substituent at the C23 carbon, proceeded smoothly, affording macrolactone **4** in high yield (Scheme 4). Finally, a phosphorylcholine moiety was attached at the free C3 hydroxy group in two successive steps to afford the desired eushearilide derivative, **3**. 

### 2.3. Antimicrobial Activity Testing

#### 2.3.1. Preparation of Antimicrobial Agents

**Figure 2:**
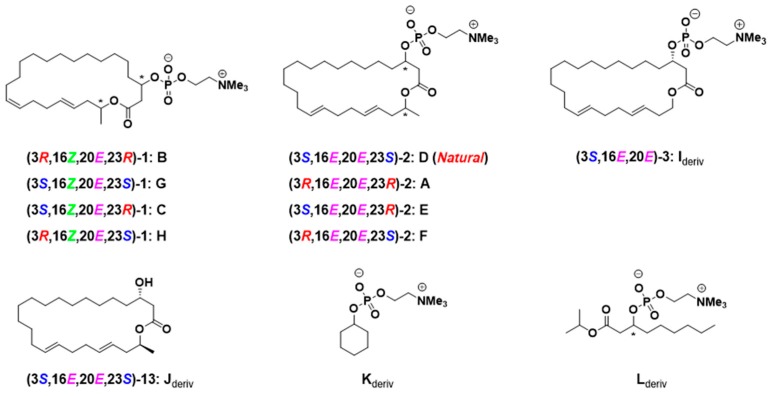
Structures and configurations of eushearilide congeners used for antimicrobial susceptibility testing.

The above describes the synthesis of the novel eushearilide congener, (3*S*,16*E*,20*E*)-**3** (I_deriv_), and eight known stereoisomers of eushearilide [9], (16*Z*,20*E*)-**1** (**B**, **G**, **C**, **H**) and (16*E*,20*E*)-**2** (**D**, **A**, **E**, **F**), each of which has different configurations corresponding to two stereogenic centers (C3, C23) and the double bond geometry (C16–C17). This preparation is shown in Figure 2. These compounds underwent antimicrobial susceptibility testing against various fungi and bacteria. Macrolactone **13** (**J**_deriv_) was also used to evaluate the effect of phosphorylcholine moieties on antimicrobial activity. Additionally, a six-membered saturated cyclic compound and an acyclic β-hydroxy ester bearing a phosphorylcholine moiety (**K**_deriv_ and **L**_deriv_; see Supplementary Materials) were prepared and investigated for comparison to eushearilide congeners.

#### 2.3.2. In Vitro Antifungal Activity

**Figure 3:**
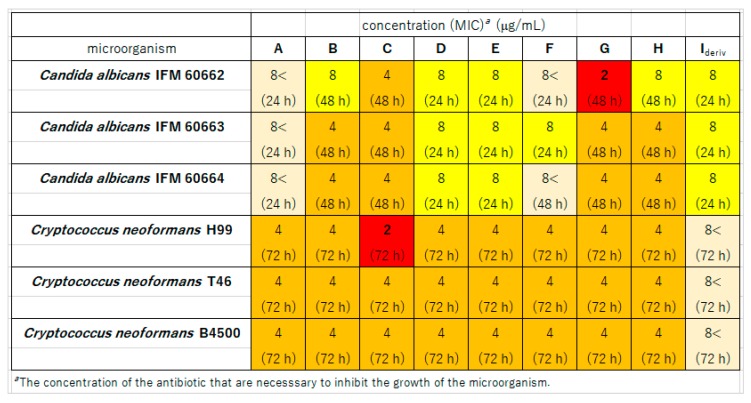
Antifungal activity of eushearilide congeners **A**–**H** and **I**_deriv_.

The antimicrobial activities of the eushearilide congeners **A**–**H** [9] and **I**_deriv_ against fungi are shown in Figure 3. Although no breakpoint was determined, eushearilide congeners **G** and **C** exhibited relatively low minimum inhibitory concentration (MIC) against *C. albicans*. In the case of *Cr. neoformans*, all compounds except eushearilide congeners **I**_deriv_ exhibited lower MIC, among which eushearilide congener **C** was the lowest.

#### 2.3.3. In Vitro Antibacterial Activity

**Figure 4:**
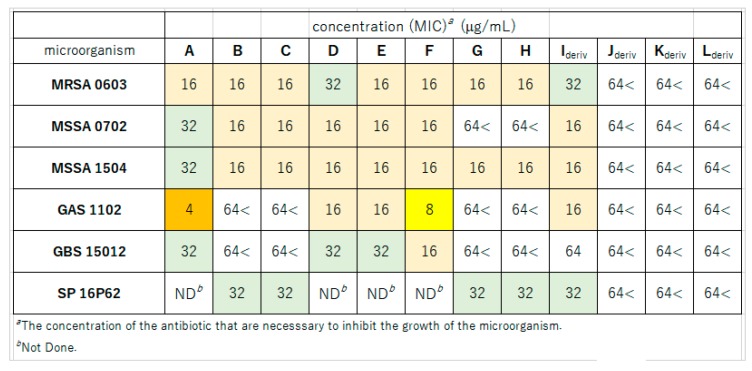
Antibacterial activity of eushearilide congeners **A**–**H**, **I**_deriv_, and related compounds.

Figure 4 shows the antibacterial activity of eushearilide congeners **A**–**H**, **I**_deriv_, and related compounds. Not all bacterial isolates were susceptible to eushearilide congener **J**_deriv_ and its related compounds. Eushearilide congeners **A**, **D**, **E**, and **F** exhibited relatively good antibacterial activity when compared to other eushearilide congeners.

## 3. Materials and Methods

### 3.1. Chemistry

#### 3.1.1. General Information

Optical rotations were determined using a Jasco P-1020 polarimeter. Infrared (IR) spectra were obtained using a Jasco FT/IR-4600 Fourier-transform infrared spectrometer. Proton and carbon nuclear magnetic resonance (^1^H and ^13^C NMR) spectra were recorded with chloroform (in CDCl_3_) on the following instrument: a JEOL JNM-AL500 (^1^H at 500 MHz and ^13^C at 125 MHz, JEOL, Tokyo, Japan). Mass spectra were determined by a Bruker Daltonics micrOTOF focus (ESI-TOF) (Bruker, Billerica, MA, USA) mass spectrometer. Thin layer chromatography was performed on a Wakogel B5F. HPLC was performed with a Hitachi LaChrom Elite system composed of an Organizer, L-2400 UV Detector, and L-2130 Pump. All reactions were carried out under an argon atmosphere in dried glassware unless otherwise noted. CH_2_Cl_2_ was distilled from diphosphorus pentoxide and then calcium hydride and dried over MS4A; toluene was distilled from diphosphorus pentoxide and dried over MS4A; and THF and diethyl ether were distilled from sodium/benzophenone immediately prior to use. All reagents were purchased from Tokyo Kasei Kogyo Co., Ltd.; Kanto Chemical Co., Inc.; or Aldrich Chemical Co., Inc.; and were used without further purification unless otherwise noted. MNBA was purchased from Tokyo Kasei Kogyo Co., Ltd. (TCI M1439).

#### 3.1.2. Experimental Procedures and Analytical Data

*(3E,7E)-21-tert-Butyldimethylsilyloxy-1-benzyloxymethoxyhenicosa-3,7-diene* (**9**): To a cooled (−78 °C) solution of sulfone **7** (120 mg, 0.225 mmol) in anhydrous DME (3.5 mL) was added KHMDS (1.0 M in THF, 0.31 mL, 0.311 mmol), and this was stirred for 5 min. Then, aldehyde **8** (42.9 mg, 0.173 mmol) in anhydrous DME (3.5 mL) was added dropwise via cold cannula to a solution for over 5 min at −78 °C. After the completion of the dripping, the mixture was stirred for 30 min. Then, the reaction mixture was quenched with H_2_O. The organic layer was separated, and the aqueous layer was extracted with diethyl ether three times. The combined organic layers were washed with brine and dried over sodium sulfate. After evaporation of the solvent, the crude product was purified by thin layer chromatography on silica gel (hexane/ethyl acetate = 8/1) to afford an *E*/*Z* mixture of **9** (75.1 mg, 78%, *E*/*Z* = 93/7) and then purified by column chromatography on silica gel impregnated with silver nitrate (hexane/ethyl acetate = 40/1) to afford **9** (67.1 mg, 70%, *E*/*Z* > 99/1). IR (neat) 2924, 2854 cm^−1^; ^1^H NMR (500 MHz, CDCl_3_) δ 7.36–7.27 (m, 5H, BOM), 5.54–5.38 (m, 4H, 3-H, 4-H, 7-H, 8-H), 4.76 (s, 2H, BOM), 4.60 (s, 2H, BOM), 3.61 (t, *J* = 6.7 Hz, 2H, 1-H), 3.60 (t, *J* = 6.7 Hz, 2H, 21-H), 2.30 (q, *J* = 6.7 Hz, 2H, 2-H), 2.05 (br s, 4H, 5-H, 6-H), 1.96 (dd, *J* = 7.2, 12.3 Hz, 2H, 9-H), 1.50 (tt, *J* = 6.7 Hz, 2H, 20-H), 1.34–1.25 (m, 20H, 10-H to 19-H), 0.894 (s, 9H, TBS), 0.05 (s, 6H, TBS); ^13^C NMR (125 MHz, CDCl_3_) δ 137.9 (BOM), 132.2 (C4), 130.8 (C8), 129.5 (C7), 128.4 (BOM), 127.8 (BOM), 127.6 (BOM), 126.4 (C3), 94.5 (BOM), 69.2 (BOM), 67.8 (C1), 63.3 (C21), 33.0 (C20), 32.9 (C2), 32.8 (C5), 32.6, 32.5 (C6, C9), 29.6, 29.6, 29.5, 29.4, 29.2 (C10 to C18), 26.0 (TBS), 25.8 (C19), 18.4 (TBS), −5.28 (TBS); HRMS (ESI/TOF) *m*/*z* [M + Na]^+^ calcd for C_35_H_62_O_3_SiNa [M + Na]^+^ 581.4366, found 581.4360.

*(14E,18E)-21-Benzyloxymethoxyhenicosa-14,18-dien-1-ol* (**10**): To a cooled (0 °C) solution of **9** (550 mg, 0.984 mmol) in THF (9.8 mL) was added TBAF (1.0 M in THF, 3.0 mL, 2.95 mmol), and this was stirred at room temperature for 2 h. After the starting material was consumed, the reaction was quenched with saturated aqueous sodium hydrogen carbonate at 0 °C. The organic layer was separated, and the aqueous layer was extracted with ethyl acetate. The combined organic layer was washed with brine and dried over sodium sulfate. After evaporation of the solvent, the crude product was purified by column chromatography on silica gel (hexane/ethyl acetate = 10/1 to 3/1) to afford **10** (418 mg, 95%). IR (neat) 3363, 2916, 2846 cm^−^^1^; ^1^H NMR (500 MHz, CDCl_3_) δ 7.36–7.27 (m, 5H, BOM), 5.54–5.35 (m, 4H, 14-H, 15-H, 18-H, 19-H), 4.76 (s, 2H, BOM), 4.60 (s, 2H, BOM), 3.62 (t, *J* = 6.8 Hz, 2H, 1-H), 3.61 (t, *J* = 6.8 Hz, 2H, 21-H), 2.30 (q, *J* = 6.7 Hz, 2H, 20-H), 2.05 (br s, 4H, 16-H,17-H), 1.98–1.94 (m, 2H, 13-H), 1.56 (qn, *J* = 6.9 Hz, 2H, 2-H), 1.33–1.26 (m, 20H, 3-H to 12-H); ^13^C NMR (125 MHz, CDCl_3_) δ 137.9 (BOM), 132.2 (C18), 130.8 (C14), 129.5 (C15), 128.3 (BOM), 127.8 (BOM), 127.6 (BOM), 126.4 (C19), 94.5 (BOM), 69.2 (BOM), 67.8 (C21), 63.0 (C1), 33.0 (C20), 32.8, 32.85 (C2, C17), 32.6, 32.5 (C13, C16), 29.6, 29.6, 29.5, 29.4, 29.1 (C4 to C12), 25.7 (C3); HRMS (ESI/TOF) *m*/*z* [M + Na]^+^ calcd for C_29_H_48_O_3_Na [M+Na]^+^ 467.3501, found 367.3502.

*(14E,18E)-21-Benzyloxymethoxyhenicosa-14,18-dienal* (**6**): To a cooled (0 °C) solution of **10** (220 mg, 0.494 mmol) in dichloromethane (4.0 mL) and DMSO (1.0 mL) were added Et_3_N (0.55 mL, 3.96 mmol) and SO_3_·Py (315 mg, 1.98 mmol). After the solution was stirred at room temperature for 2.5 h, the reaction mixture was quenched with saturated aqueous ammonium chloride at 0 °C. The organic layer was separated, and the aqueous layer was extracted with ethyl acetate. The combined organic layer was washed with brine and dried over sodium sulfate. After evaporation of the solvent, the crude product was purified by thin layer chromatography on silica gel (hexane/ethyl acetate = 5/1) to afford **6** (191 mg, 87%). IR (neat) 3425, 2916, 2846, 1712 cm^−^^1^; ^1^H NMR (500 MHz, CDCl_3_) δ 9.76 (t, *J* = 1.7 Hz, 1H, 1-H), 7.39–7.27 (m, 5H, BOM), 5.55–5.35 (m, 4H, 14-H, 15-H, 18-H, 19-H), 4.76 (s, 2H, BOM), 4.60 (s, 2H, BOM), 3.60 (t, *J* = 6.9 Hz, 2H, 21-H), 2.41 (td, *J* = 1.7, 7.4 Hz, 2H, 2-H), 2.30 (q, *J* = 6.5 Hz, 2H, 20-H), 2.05 (br s, 4H, 16-H, 17-H), 1.98–1.94 (m, 2H, 13-H), 1.62 (tt, *J* = 7.3 Hz, 2H, 3-H), 1.32–1.27 (m, 18H, 4-H to 12-H); ^13^C NMR (125 MHz, CDCl_3_): δ 202.9 (C1), 137.9 (BOM), 132.2 (C18), 130.8 (C14), 129.5 (C15), 128.4 (BOM), 127.8 (BOM), 127.6 (BOM), 126.4 (C19), 94.5 (BOM), 69.2 (BOM), 67.8 (C21), 43.9 (C2), 33.0 (C20), 32.8 (C17), 32.6, 32.5 (C13, C16), 29.6, 29.5, 29.5, 29.4, 29.3, 29.1 (C4 to C12), 22.1 (C3); HRMS (ESI/TOF) *m*/*z* [M+Na]^+^ calcd for C_29_H_46_O_3_Na [M + Na]^+^ 465.3345, found 465.3330.

*(3S,16E,20E)-Ethyl 23-benzyloxymethoxy-3-hydroxytricosa-16,20-dienethioate* (**11**): To a solution of Sn(OTf)_2_ (268 mg, 0.643 mmol) in dichloromethane (4.4 mL) were added solutions of (*R*)-1-methyl-2-(1-naphthylaminomethyl)pyrrolidine (168 mg, 0.700 mmol) in dichloromethane (1.4 mL) and *^n^*Bu_3_SnF (199 mg, 0.644 mmol). The mixture was cooled to −78 °C. To the reaction mixture were added solutions of KSA (114 mg, 0.643 mmol) in dichloromethane (1.4 mL) and the aldehyde **6** (190 mg, 0.429 mmol) in dichloromethane (1.0 mL) at −78 °C, successively. The mixture was stirred for 1 h at that temperature and then quenched with saturated aqueous sodium hydrogen carbonate. The organic layer was separated, and the aqueous layer was extracted with dichloromethane three times. The combined organic layer was washed with brine and dried over sodium sulfate. After evaporation of the solvent, the crude product was purified by thin layer chromatography on silica gel (hexane/ethyl acetate = 3/1) to afford the aldol product **11** (187 mg, 80%, dr = 96/4). ${\left[\alpha \right]}_{24}^{D}$ +9.06 (*c* 0.78, CHCl_3_); IR (neat) 3464, 3433, 2924, 2854, 1682 cm^−1^; ^1^H NMR (500 MHz, CDCl_3_): δ 7.38–7.27 (m, 5H, BOM), 5.55–5.35 (m, 4H, 16-H, 17-H, 20-H, 21-H), 4.76 (s, 2H, BOM), 4.60 (s, 2H, BOM), 4.06–4.02 (m, 1H, 3-H), 3.60 (t, *J* = 6.9 Hz, 2H, 23-H), 2.90 (q, *J* = 7.4 Hz, 2H, Et), 2.73 (dd, *J* = 3.4, 15.5 Hz, 1H, 2-H), 2.65 (dd, *J* = 8.6, 15.5 Hz, 1H, 2-H), 2.30 (q, *J* = 6.7 Hz, 1H, 22-H), 2.04 (br s, 4H, 18-H, 19-H), 1.98–1.94 (m, 2H, 15-H), 1.53–1.39 (m, 4H, 4-H, 5-H), 1.32–1.26 (m, 21H, 6-H to 14-H, Et); ^13^C NMR (125 MHz, CDCl_3_) δ 199.7 (C1), 137.9 (BOM), 132.2 (C20), 130.8 (C16), 129.5 (C17), 128.4 (BOM), 127.9 (BOM), 127.6 (BOM), 126.4 (C21), 94.5 (BOM), 69.3 (BOM), 68.7 (C3), 67.8 (C23), 50.6 (C2), 36.5 (C4), 33.0 (C22), 32.8 (C19), 32.6, 32.5 (C15, C18), 29.6, 29.6, 29.5, 29.5, 29.5, 29.2 (C6 to C14), 25.4 (C5), 23.4 (Et), 14.6 (Et); HRMS (ESI/TOF) *m*/*z* [M + Na]^+^ calcd for C_33_H_54_O_4_SNa [M + Na]^+^ 569.3635, found 569.3640.

*(3S,16E,20E)-Ethyl 23-benzyloxymethoxy-3-hydroxytricosa-16,20-dienoate* (**12**): To a cooled (0 °C) solution of **11** (172 mg, 0.315 mmol) in EtOH (3.1 mL) were added *^i^*Pr_2_NEt (0.22 mL, 1.26 mmol) and AgOCOCF_3_ (139 mg, 0.629 mmol). After the solution was stirred at room temperature for 7 h, the reaction mixture was filtered through Celite. After evaporation of the solvent, the crude product was purified by thin layer chromatography on silica gel (hexane/ethyl acetate = 3/1) to afford **57** (168 mg, quant.). ${\left[\alpha \right]}_{24}^{D}$ +6.91 (*c* 1.03, CHCl_3_); IR (neat) 3433, 2916, 2846, 1712 cm^−1^; ^1^H NMR (500 MHz, CDCl_3_) δ 7.36–7.28 (m, 5H, BOM), 5.54–5.35 (m, 4H, 16-H, 17-H, 20-H, 21-H), 4.76 (s, 2H, BOM), 4.60 (s, 2H, BOM), 4.17 (q, *J* = 7.3 Hz, 2H, Et), 3.99 (brs, 1H, 3-H), 3.60 (t, *J* = 6.9, 2H, 23-H), 2.93 (s, 1H, OH), 2.50 (dd, *J* = 2.9, 16.6 Hz, 1H, 2-H), 2.39 (dd, *J* = 9.2, 16.6 Hz, 1H, 2-H), 2.30 (q, *J* = 6.5 Hz, 2H, 22-H), 2.04 (br s, 4H, 18-H, 19-H), 1.96 (dd, *J* = 7.4, 12.0 Hz, 2H, 15-H), 1.53–1.39 (m, 4H, 4-H, 5-H), 1.32–1.25 (m, 21H, 6-H to 14-H, Et); ^13^C NMR (125 MHz, CDCl_3_) δ 173.0 (C1), 137.9 (BOM), 132.1 (C20), 130.8 (C16), 129.4 (C17), 128.3 (BOM), 127.8 (BOM), 127.6 (BOM), 126.4 (C21), 94.5 (BOM), 69.2 (BOM), 68.0 (C3), 67.8 (C23), 60.6 (Et), 41.3 (C2), 36.5 (C4), 33.0 (C22), 32.7 (C19), 32.5, 32.5 (C15, C18), 29.6, 29.6, 29.5, 29.5, 29.1 (C6 to C14), 25.4 (C5), 14.1 (Et); HRMS (ESI/TOF) *m*/*z* [M + Na]^+^ calcd for C_33_H_54_O_5_Na [M + Na]^+^ 553.3869, found 553.3866.

*(3S,16E,20E)-3,23-Dihydroxytricosa-16,20-dienoic acid* (**5**): A solution of **12** (168 mg, 0.317 mmol) in 2.0 M HCl (EtOH/HCl = 5/1, 6.3 mL) was stirred at room temperature for 10 h. After the starting material was consumed, 4.0 M aqueous LiOH was added at 0 °C. Distilled water (0.91 mL) was poured into the reaction mixture, followed by the addition of 4.0 M aqueous LiOH (0.16 mL, 0.633 mmol). While being stirred at room temperature for 20 h, 1.0 M aqueous HCl was added until the solution became acidic. The organic layer was separated, and the aqueous layer was extracted with ethyl acetate five times. The combined organic layer was washed with water and brine and dried over sodium sulfate. After evaporation of the solvent, the organic phase was added (10% aqueous NaOH solution) and washed with ethyl acetate five times. The aqueous phase was separated, 1.0 M aqueous HCl solution was added, and it was washed with ethyl acetate five times. The combined organic layers were washed with water and brine and dried over sodium sulfate. After evaporation of the solvent, the desired product **5** (115 mg, 95%) was obtained as a white solid. M.P. 81.3–82.1 °C; ${\left[\alpha \right]}_{25}^{D}$ −2.44 (*c* 0.23, CH_3_OH); IR (neat): 3402, 3379, 2916, 2846 cm^−1^; ^1^H NMR (500 MHz, CD_3_OD) δ 5.47–5.39 (m, 4H, 16-H, 17-H, 20-H, 21-H), 3.98–3.93 (m, 1H, 3-H), 3.52 (t, *J* = 6.9 Hz, 23-H), 2.43 (dd, *J* = 4.9, 15.2 Hz, 1H, 2-H), 2.35 (dd, *J* = 8.3, 15.2 Hz, 1H, 2-H), 2.20 (q, *J* = 6.7 Hz, 2H, 22-H), 2.04 (br s, 4H, 18-H, 19-H), 1.97 (dd, *J* = 6.9, 11.5 Hz, 2H, 15-H), 1.47–1.46 (m, 4H, 4-H, 5-H), 1.33–1.28 (m, 18H, 6-H to 14-H); ^13^C NMR (125 MHz, CD_3_OD) δ 175.9 (C1), 133.3 (C20), 131.9 (C16), 130.9 (C17), 127.8 (C21), 69.4 (C3), 63.1 (C23), 43.4 (C2), 38.1 (C4), 37.1 (C22), 34.0 (C19), 33.7, 33.6 (C15, C18), 30.8, 30.8, 30.6, 30.2 (C6 to 14), 26.7 (C5); HRMS (ESI/TOF) *m*/*z* [M + Na]^+^ calcd for C_23_H_42_O_4_Na [M + Na]^+^ 405.2981, found 405.2979.

*(3S,16E,20E)-3-Hydroxytricosa-16,20-dienolide* (**4**): To a solution of MNBA (65.5 mg, 0.190 mmol) and DMAP (107 mg, 0.878 mmol) in dichloromethane (58 mL) at room temperature was slowly added a solution of the seco acid **5** (56.0 mg, 146 mmol) in dichloromethane (15 mL) with a mechanically driven syringe over a 12 h period. After cooling to 0 °C, saturated aqueous sodium hydrogen carbonate was added. The organic layer was separated, the aqueous layer was extracted with dichloromethane, and the organic layer was washed with brine and dried over sodium sulfate. After evaporation of the solvent, the crude product was purified by thin layer chromatography on silica gel (hexane/ethyl acetate = 3/1) to afford **41** (45.6 mg, 85%). ${\left[\alpha \right]}_{24}^{D}$ +9.71 (*c* 0.97, CHCl_3_); IR (neat) 3448, 2924, 2854, 1728 cm^−^^1^; ^1^H NMR (500 MHz, CDCl_3_): δ 5.53–5.37 (m, 4H, 16-H, 17-H, 20-H, 21-H), 4.20–4.10 (m, 2H, 23-H), 3.96 (brs, 1H, 3-H), 2.79 (s, 1H, OH), 2.52 (dd, *J* = 4.0, 15.5 Hz, 1H, 2-H), 2.42 (dd, *J* = 7.7, 15.8 Hz, 1H, 2-H), 2.33 (q, *J* = 5.9 Hz, 2H, 22-H), 2.05 (br s, 4H, 18-H, 19-H), 1.99 (dd, *J* = 6.3, 10.3 Hz, 2H, 15-H), 1.54–1.42 (m, 2H, 4-H), 1.39–1.27 (m, 20 H, 5-H to 14-H); ^13^C NMR (125 MHz, CDCl_3_) δ 172.6 (C1), 133.6 (C20), 130.8 (C16), 129.8 (C17), 125.7 (C21), 68.1 (C3), 63.9 (C23), 41.3 (C2), 36.1 (C4), 32.8 (C18), 32.5 (C19), 32.0, 31.9 (C15, C22), 28.6, 28.5, 28.4, 28.3, 28.2, 28.1, 28.0, 27.4 (C6 to C14), 24.7 (C5); HRMS (ESI/TOF) *m*/*z* [M + Na]^+^ calcd for C_23_H_40_O_3_Na [M + Na]^+^ 387.2875, found 387.2868.

*23-Demethyleushearilide* (**3**) (**I**_deriv_): To a cooled (0 °C) solution of **4** (45 mg, 0.123 mmol) in toluene (2.5 mL) were added Et_3_N (69 μL, 0.493 mmol) and 2-chloro-2-oxo-1,3,2-dioxaphospholane (34 μL, 0.370 mmol). After the solution was stirred at room temperature for 4.5 h, the crude product obtained after the separation of amine salt by filtration was used in the next step without further purification. To a cooled (−15 °C) solution of the crude product in acetonitrile (2.5 mL) was added an excess amount of Me_3_N. The solution was stirred at 70 °C for 14 h in a stirred autoclave of stainless steel. After cooling to room temperature, the solvent was removed, and the crude product was purified by thin layer chromatography on silica gel (Chromatorex NH-DM1020; chloroform/MeOH = 6/1) to afford 23-demethyleushearilide (**3**) (23.9 mg, 37%). ${\left[\alpha \right]}_{24}^{D}$ +8.66 (*c* 1.14, CH_3_OH); IR (neat) 3433, 3402, 2924, 2862, 1728 cm^−1^; ^1^H NMR (500 MHz, CD_3_OD) δ 5.49–5.37 (m, 4H, 16-H, 17-H, 20-H, 21-H), 4.56-4.53 (m, 1H, 3-H), 4.26 (m, 2H, 24-H), 4.12–4.03 (m, 2H, 23-H), 3.63–3.62 (m, 2H, 25-H), 3.22 (s, 9H, 26-H), 2.80 (dd, *J* = 4.6, 14.9 Hz, 1H, 2-H), 2.56 (dd, *J* = 8.0, 14.9 Hz, 1H, 2-H), 2.31 (q, 2H, *J* = 6.5 Hz, 22-H), 2.06 (br s, 4H, 18-H, 19-H), 2.00 (dd, *J* =5.4, 11.7 Hz, 2H, 15-H), 1.68–1.63 (m, 2H, 4-H), 1.44–1.30 (m, 20H, 5-H to 14-H); ^13^C NMR (125 MHz, CD_3_OD) δ 172.4 (C1), 134.0 (C20), 131.8 (C16), 131.2 (C17), 127.0 (C21), 74.1 (d, *J* = 6.0 Hz, C3), 67.5 (C25), 65.3 (C23), 60.3 (d, *J* = 6.0 Hz, C24), 54.7 (t, *J* = 3.6 Hz, C26), 41.4 (d, *J* = 2.4 Hz, C2), 36.2 (d, *J* = 4.8 Hz, C4), 34.0, 33.6 (C18, C19), 33.1 (C20), 32.7 (C15), 30.1 (C6), 29.8, 29.8, 29.7, 29.6, 29.5, 29.4, 29.2, 28.3 (C7 to C14), 25.4 (C5); HRMS (ESI/TOF) *m*/*z* [M + Na]^+^ calcd for C_28_H_52_NO_6_PNa [M + Na]^+^ 552.3430, found 552.3446.

Phosphate **K**_deriv_: To a cooled (0 °C) solution of cyclohexanol (30 mg, 0.30 mmol) in toluene (3.0 mL) were added Et_3_N (71 μL, 0.51 mmol) and 2-chloro-2-oxo-1,3,2-dioxaphospholane (36 μL, 0.39 mmol). After the solution was stirred at room temperature for 2.5 h, the crude product obtained after the separation of amine salt by filtration was used in the next step without further purification. To a cooled (−15 °C) solution of the crude product in acetonitrile (3.0 mL) was added an excess amount (ca. 20 eq.) of Me_3_N. The solution was stirred at 70 °C for 14 h in a stirred autoclave of stainless steel. After cooling to room temperature, the solvent was removed, and the crude product was purified by thin layer chromatography on silica gel (Chromatorex NH-DM1020; chloroform/MeOH = 5/1) to afford the desired product (**K**_deriv_) (36.9 mg, 46%). IR (neat) 2931, 2854, 1226, 1087, 1049 cm^−1^; ^1^H NMR (500 MHz, CD_3_OD) *δ* 4.26–4.21 (m, 2H, 7-H), 4.17–4.09 (m, 1H, 1-H), 3.64–3.59 (m, 2H, 8-H), 3.21 (s, 9H, 9-H), 1.98–1.91 (m, 2H, 2-H, 6-H), 1.78–1.71 (m, 2H, 2-H, 6-H), 1.56–1.20 (m, 6H, 3-H to 4-H); ^13^C NMR (125 MHz, CD_3_OD) δ 75.8 (d, *J* = 6.0 Hz), 67.6–67.4 (m), 60.2 (d, *J* = 4.8 Hz), 54.8–54.6 (m), 34.9 (d, *J* = 3.6 Hz), 26.5, 24.9; HRMS (ESI/TOF) *m*/*z* [M + Na]^+^ calcd for C_11_H_24_NO_4_PNa 288.1335, found 288.1328.

Phosphate **L**_deriv_: To a cooled (0 °C) solution of isopropyl 3-hydroxynonanoate (40 mg, 0.18 mmol) in toluene (3.7 mL) were added Et_3_N (0.13 mL, 0.92 mmol) and 2-chloro-2-oxo-1,3,2-dioxaphospholane (68 μL, 0.74 mmol). After the solution was stirred at room temperature for 3 h, the crude product obtained after the separation of amine salt by filtration was used in the next step without further purification. To a cooled (−15 °C) solution of the crude product in acetonitrile (3.7 mL) was added an excess amount (ca. 20 eq.) of Me_3_N. The solution was stirred at 70 °C for 15 h in a stirred autoclave of stainless steel. After cooling to room temperature, the solvent was removed, and the crude product was purified by thin layer chromatography on silica gel (Chromatorex NH-DM1020; chloroform/MeOH = 5/1) to afford the desired product (**L**_deriv_) (32.3 mg, 46%). M.P. 164.3–164.9 °C; IR (KBr) 2931, 2862, 1720, 1643, 1227, 1088 cm^−1^; ^1^H NMR (500 MHz, CD_3_OD); *δ* 4.96 (sep, *J* = 6.0 Hz, 1H, *^i^*Pr CH), 4.52 (dtd, *J* = 13.0, 6.5, 6.5 Hz, 1H, 3-H), 4.25 (m, 2H, 10-H), 3.62 (m, 2H, 11-H), 3.22 (s, 9H, 12-H), 2.73 (dd, *J* =15.0, 6.0 Hz, 1H, 2-H), 2.52 (dd, *J* =15.0, 7.0 Hz, 1H, 2-H), 1.71–1.60 (m, 2H, 4-H), 1.47–1.26 (m, 8H, 5-H to 8-H), 1.23 (d, *J* = 2.0 Hz, 3H, *^i^*Pr CH_3_), 1.22 (d, *J* = 5.9 Hz, 3H, *^i^*Pr CH_3_), 0.90 (t, *J* = 7.0 Hz, 3H, 9-H); ^13^C NMR (125 MHz, CD_3_OD) δ 172.1, 74.3 (d, *J* = 5.9 Hz), 69.1, 67.5, 60.3 (d, *J* = 4.8 Hz), 54.7, 41.8 (d, *J* = 3.6 Hz), 36.6 (d, *J* = 3.5 Hz), 32.9, 30.4, 26.0, 23.7, 22.11, 22.07, 14.4; HRMS (ESI/TOF) *m*/*z* [M+Na]^+^ calcd for C_17_H_37_NO_6_PNa 382.2353, found 382.2360.

### 3.2. Biology

#### 3.2.1. Materials

Six gram-positive bacterial strains were randomly selected from stocks in the Medical Mycology Research Center, Chiba University. All strains were clinical isolates and were included in methicillin-sensitive *Staphylococcus aureus*, methicillin-resistant *S. aureus*, *Streptococcus pyogenes*, *Streptococcus agalactiae*, and *Streptococcus pneumoniae*. *Candida albicans* and *Cryptococcus neoformans* were used for testing antifungal activity. Three isolates from each species were used, all of which were stored and maintained in the Culture Collection of the Medical Mycology Research Center, Chiba University.

#### 3.2.2. Method for In Vitro Antifungal Assay

Antifungal activities were primarily determined based on a slightly modified M27A3 reference method for the microdilution by CLSI. All eushearilide congeners **A**–**H, I**_deriv_, and their related compounds, diluted with ethanol, had distilled water added to them, and then RPMI1640, for adjustment to the desired concentrations. Yeasts were cultured on Sabouraud dextrose agar, collected, and suspended in RPMI 1640. Concentrations were adjusted using RPMI 1640 and then mixed with eushearilide congeners **A**–**H**, **I**_deriv_, and their related compounds to achieve the final concentration of 1 × 10^3^ cfu/mL of yeast. Yeast with each compound was cultured at 35 °C for 24 to 48 h, and the MICs were determined using IC_50_ as the endpoint with a reading mirror.

#### 3.2.3. Method for In Vitro Antibacterial Assay

After each bacterial strain was suspended in the sterilized saline solution, the bacterial suspension was adjusted using the McFarland 0.5 standard. The adjusted bacterial suspension was further diluted 10 times using sterilized saline solution before being added to each 5 μL/well in a 96-well plate. The eushearilide congeners **A**–**H**, **I**_deriv_, and their related compounds were initially diluted using ethanol and then distilled water. Two-fold dilution series from eight-folds 5 dilutions up were added to 100 μL/well in a 96-well plate. A Mueller Hinton bouillon was used for dilution. The testing of *St. pyogenes*, *St. agalactiae*, and *St. pneumoniae* required an additional hemo-supplement for dilution. Minimal inhibitory concentration was measured after 22–24 h incubation in a 35 °C incubator.

## 4. Conclusions

The seven stereoisomers of eushearilide and its 23-demethyl congener were synthesized through a convergent reaction process, which mainly featured the Julia–Kocienski olefination, the asymmetric Mukaiyama aldol reaction, and MNBA-mediated macrolactonization based on our established synthesis of naturally occurring eushearilide. These eushearilide congeners were subjected to antimicrobial activity assays against a broad range of fungi and bacteria. Two of the eushearilide congeners examined appeared to be active against *C. albicans*, whereas most of the congeners expressed activity against *Cr. neoformans*. The eushearilide congeners **A**, **D**, **E**, and **F** had relatively good antibacterial activity against gram-positive bacteria, including methicillin-resistant *S. aureus*. Thus, eushearilides were found to exhibit a wide range of antimicrobial activity not only against fungi but also against bacteria, including methicillin-resistant *Staphylococcus aureus* (MRSA) and vancomycin-resistant enterococci (VRE).

## Supplementary Materials

The Supplementary Materials comprise copies of ^1^H and ^13^C NMR spectra for all new compounds, and additional data (schemes of phosphates **K**_deriv_ and **L**_deriv_) are available online.

## References

[1] White T.C., Marr K.A., Bowden R.A. (1998). Clinical, Cellular, and Molecular Factors That Contribute to Antifungal Drug Resistance. Clin. Microbiol. Rev..

[2] Ghannoum M.A., Rice L.B. (1999). Antifungal Agents: Mode of Action, Mechanisms of Resistance, and Correlation of These Mechanisms with Bacterial Resistance. Clin. Microbiol. Rev..

[3] Rychnovsky S.D. (1995). Oxo Polyene Macrolide Antibiotics. Chem. Rev..

[4] Rex J.H., Rinaldi M.G., Pfaller M.A. (1995). Resistance of *Candida* Species to Fluconazole. Antimicrob. Agents Chemother..

[5] Hosoe T., Fukushima K., Takizawa K., Itabashi T., Kawahara N., Vidotto V., Kawai K. (2006). A New Antifungal Macrolide, Eushearilide, Isolated from *Eupenicillium shearii*. J. Antibiot..

[6] Tonoi T., Kawahara R., Yoshinaga Y., Inohana T., Fujimori K., Shiina I. (2015). Total Synthesis of (3*R*,16*E*,20*E*,23*R*)-(−)-Eushearilide and Structural Determination of Naturally Occurring Eushearilide. Tetrahedron Lett..

[7] Yamauchi T., Takidaira J., Okamoto K., Sugiura T., Horikoshi H., Kudo S., Sasaki S., Mizushima N., Higashiyama K. (2014). Total synthesis of the proposed structures of Eushearilide. Heterocycles.

[8] Tonoi T., Kawahara R., Inohana T., Shiina I. (2016). Enantioselective Total Synthesis of Naturally Occurring Eushearilide and Evaluation of Its Antifungal Activity. J. Antibiot..

[9] Tonoi T., Inohana T., Sato T., Yoshida T., Shiina I. (2018). Total Synthesis and Antimicrobial Activities of All Stereoisomers of (16*Z*,20*E*)-Eushearilide and (16*E*,20*E*)-Eushearilide. J. Org. Chem..

[10] Qin D., Byun H.-S., Bittman R. (1999). Synthesis of Plasmalogen via 2,3-Bis-*O*-(4′-methoxybenzyl)-*sn*-glycerol. J. Am. Chem. Soc..

[11] Shiina I., Ibuka R., Kubota M. (2002). A New Condensation Reaction for the Synthesis of Carboxylic Esters from Nearly Equimolar Amounts of Carboxylic Acids and Alcohols Using 2-Methyl-6-nitrobenzoic Anhydride. Chem. Lett..

[12] Shiina I., Kubota M., Ibuka R. (2002). A Novel and Efficient Macrolactonization of ω-Hydroxycarboxylic Acids Using 2-Methyl-6-Nitrobenzoic Anhydride (MNBA). Tetrahedron Lett..

[13] Shiina I., Kubota M., Oshiumi H., Hashizume M. (2004). An Effective Use of Benzoic Anhydride and Its Derivatives for the Synthesis of Carboxylic Esters and Lactones: A Powerful and Convenient Mixed Anhydride Method Promoted by Basic Catalysts. J. Org. Chem..

[14] Shiina I. (2014). An Adventurous Synthetic Journey with MNBA from Its Reaction Chemistry to the Total Synthesis of Natural Products. Bull. Chem. Soc. Jpn..

[15] Shiina I. (2007). Total Synthesis of Natural 8- and 9-Membered Lactones: Recent Advancements in Medium-Sized Ring Formation. Chem. Rev..

[16] Mukaiyama T., Kobayashi S., Uchiro H., Shiina I. (1990). Catalytic Asymmetric Aldol Reaction of Silyl Enol Ethers with Aldehydes by the Use of Chiral Diamine Coordinated Tin(II) Triflate. Chem. Lett..

[17] Kobayashi S., Uchiro H., Fujishita Y., Shiina I., Mukaiyama T. (1991). Asymmetric Aldol Reaction between Achiral Silyl Enol Ethers and Achiral Aldehydes by Use of a Chiral Promoter System. J. Am. Chem. Soc..

[18] Shiina I. (2014). Asymmetric Mukaiyama Aldol Reactions Using Chiral Diamine–Coordinated Sn(II) Triflate: Development and Application to Natural Product Synthesis. Chem. Rec..

[19] Blakemore P.R., Cole W.J., Kocienski P.J., Morley A. (1998). A Stereoselective Synthesis of *trans*-1,2-Disubstituted Alkenes Based on the Condensation of Aldehydes with Metallated 1-Phenyl-1*H*-tetrazol-5-yl Sulfones. Synlett.

[20] Aïssa C. (2009). Mechanistic Manifold and New Developments of the Julia–Kocienski Reaction. Eur. J. Org. Chem..

[21] Blakemore P.R. (2002). The Modified Julia Olefination: Alkene Synthesis via the Condensation of Metallated Heteroarylalkylsulfones with Carbonyl Compounds. J. Chem. Soc. Perkin Trans. 1.

