# Supplementary material for: Total Synthesis and Antimicrobial Evaluation of 23-Demethyleushearilide and Extensive Antimicrobial Evaluation of All Synthetic Stereoisomers of (16Z,20E)-Eushearilide and (16E,20E)-Eushearilide
Source: Molecules. 2019 Sep 22;24(19):3437. doi: 10.3390/molecules24193437 (PMC6804234; doi:10.3390/molecules24193437)
Supplement: Supplementary file 1 [file molecules-24-03437-s001.pdf]

Supplementary Materials

Total Synthesis and Antimicrobial Evaluation of 23-Demethyleushearilide and Extensive Antimicrobial Evaluation of All Synthetic Stereoisomers of (16*Z*,20*E*)-Eushearilide and (16*E*,20*E*)-Eushearilide

Takayuki Tonoi [1,*], Takehiko Inohana [1], Teruyuki Sato [1], Yuuki Noda [1], Miyuki Ikeda [1], Miku Akutsu [1], Takatsugu Murata [1], Yutaro Maekawa [1], Anna Tanaka [1], Rio Seki [2], Misako Ohkusu [2], Katsuhiko Kamei [2,*], Naruhiko Ishiwada [2,*] and Isamu Shiina [1,*]

[1]Department of Applied Chemistry, Faculty of Science, Tokyo University of Science, 1-3 Kagurazaka, Shinjuku-ku, Tokyo 162-8601, Japan

[2]Department of Infectious Diseases, Medical Mycology Research Center, Chiba University, 1-8-1 Inohana, Chuo-ku, Chiba-shi, Chiba 260-8673, Japan

Table of contents

1. Preparation of Phosphates $\mathbf{K}_{deriv}$ and $\mathbf{L}_{deriv}$: S2
2. Copies of ^{1}H and ^{13}C NMR spectra: S3 to S24

1. Preparation of Phosphates **K**deriv and **L**deriv

Phosphate **K**deriv was prepared as shown below.

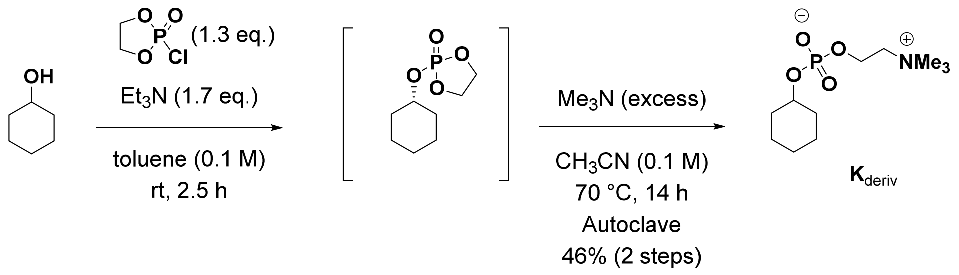

Phosphate **L**deriv was prepared as shown below.

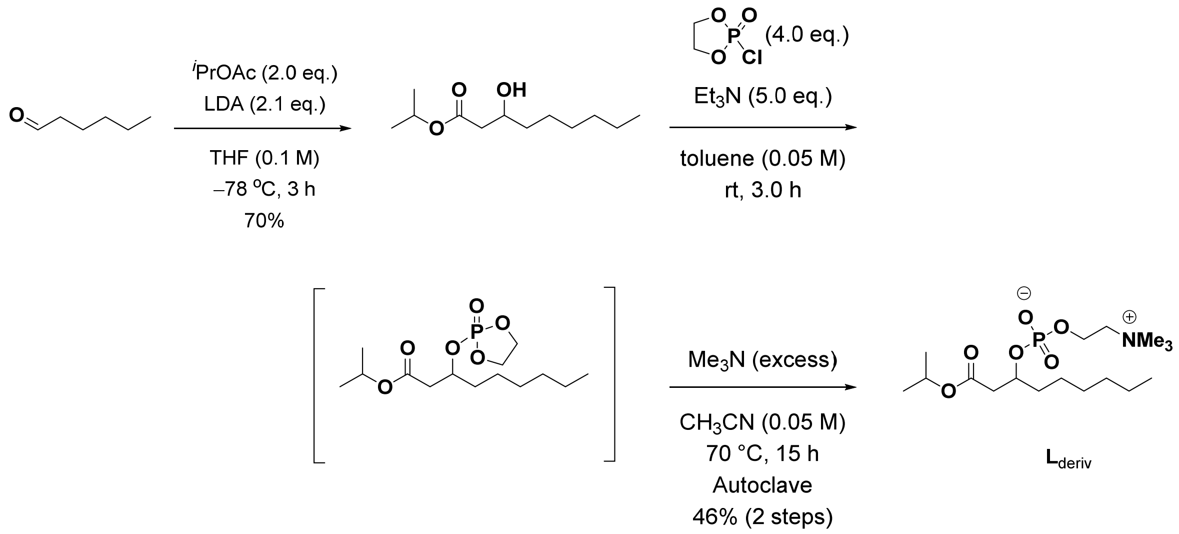

single_pulse

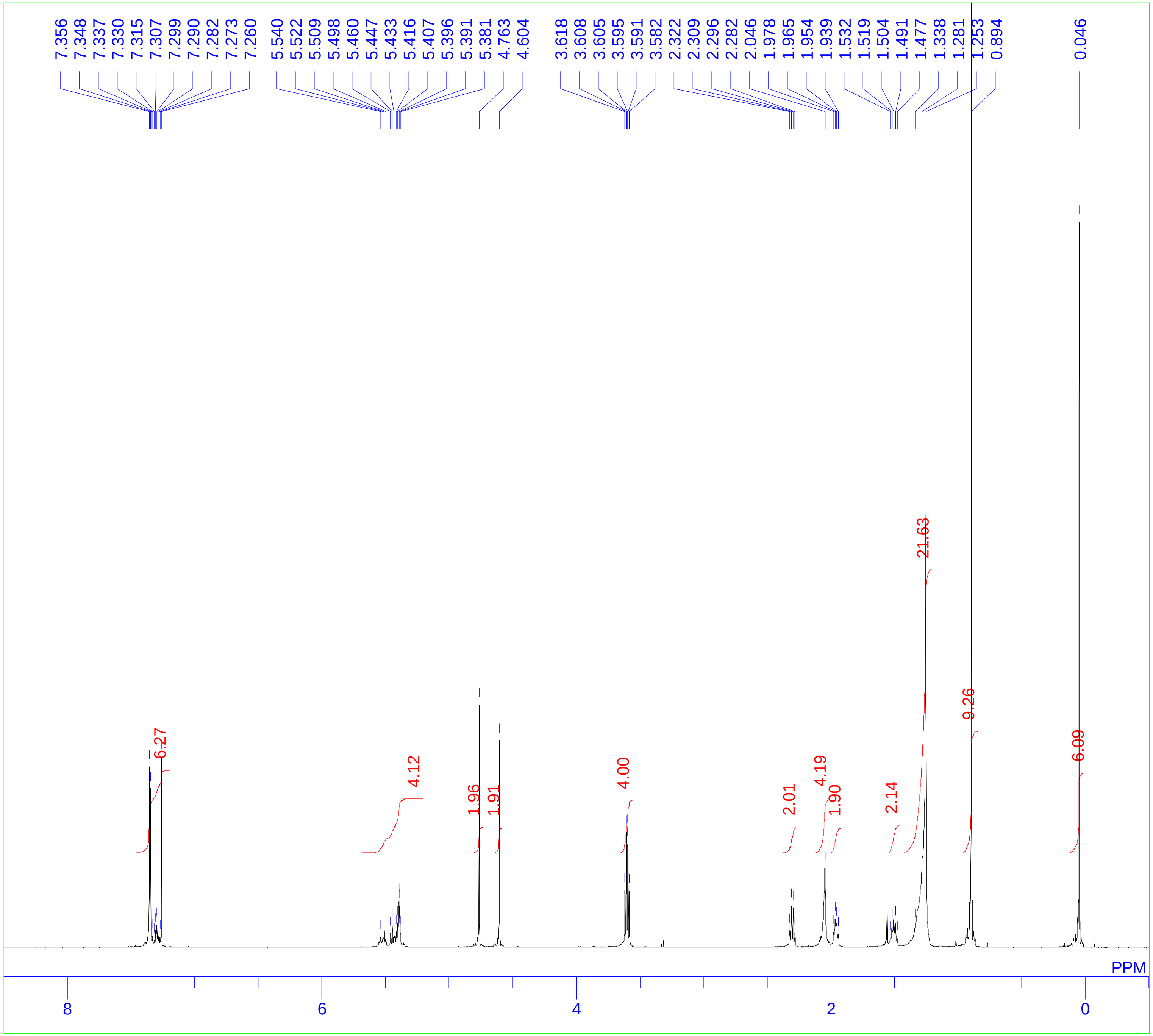

DFILE	IT-3-002-E_Proton-1-2.als
COMNT	single_pulse
DATIM	2016-07-27 17:19:21
OBNUC	1H
EXMOD	proton.jxp
OBFRQ	500.16 MHz
OBSET	2.41 KHz
OBFIN	6.01 Hz
POINT	16384
FREQU	9384.38 Hz
SCANS	8
ACQTM	1.7459 sec
PD	5.0000 sec
PW1	3.52 usec
IRNUC	1H
CTEMP	23.0 c
SLVNT	CDCL3
EXREF	7.26 ppm
BF	0.12 Hz
RGAIN	42

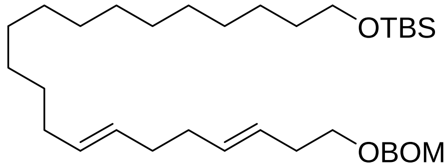

(3*E*,7*E*)-21-*tert*-Butyldimethylsilyloxy-1-benzyloxymethoxyhenicosa-3,7-diene (**9**)

single pulse decoupled gated NOE

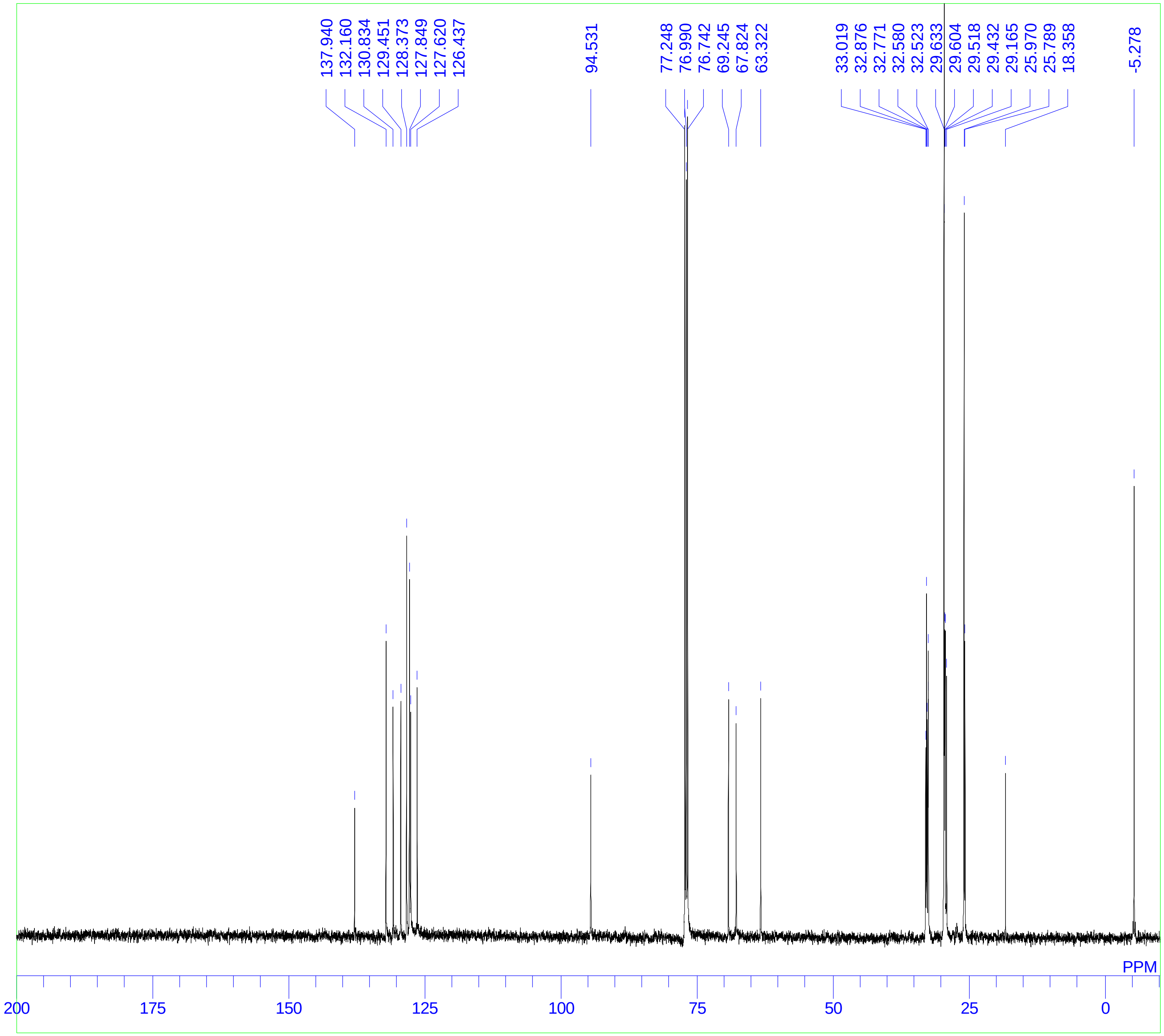

DFILE	IT-3-014-E_Carbon-1-2.als
COMNT	single pulse decoupled gated NOE
DATIM	2016-08-06 11:27:20
OBNUC	13C
EXMOD	carbon.jxp
OBFRQ	125.77 MHz
OBSET	7.87 KHz
OBFIN	4.21 Hz
POINT	32768
FREQU	39308.18 Hz
SCANS	532
ACQTM	0.8336 sec
PD	2.0000 sec
PW1	3.74 usec
IRNUC	1H
CTEMP	23.1 c
SLVNT	CDCL3
EXREF	77.00 ppm
BF	0.12 Hz
RGAIN	24

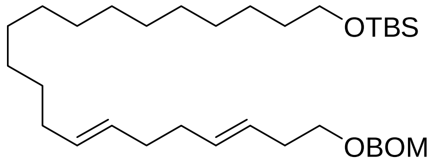

(3*E*,7*E*)-21-*tert*-Butyldimethylsilyloxy-1-benzyloxymethoxyhenicosa-3,7-diene (**9**)

single_pulse

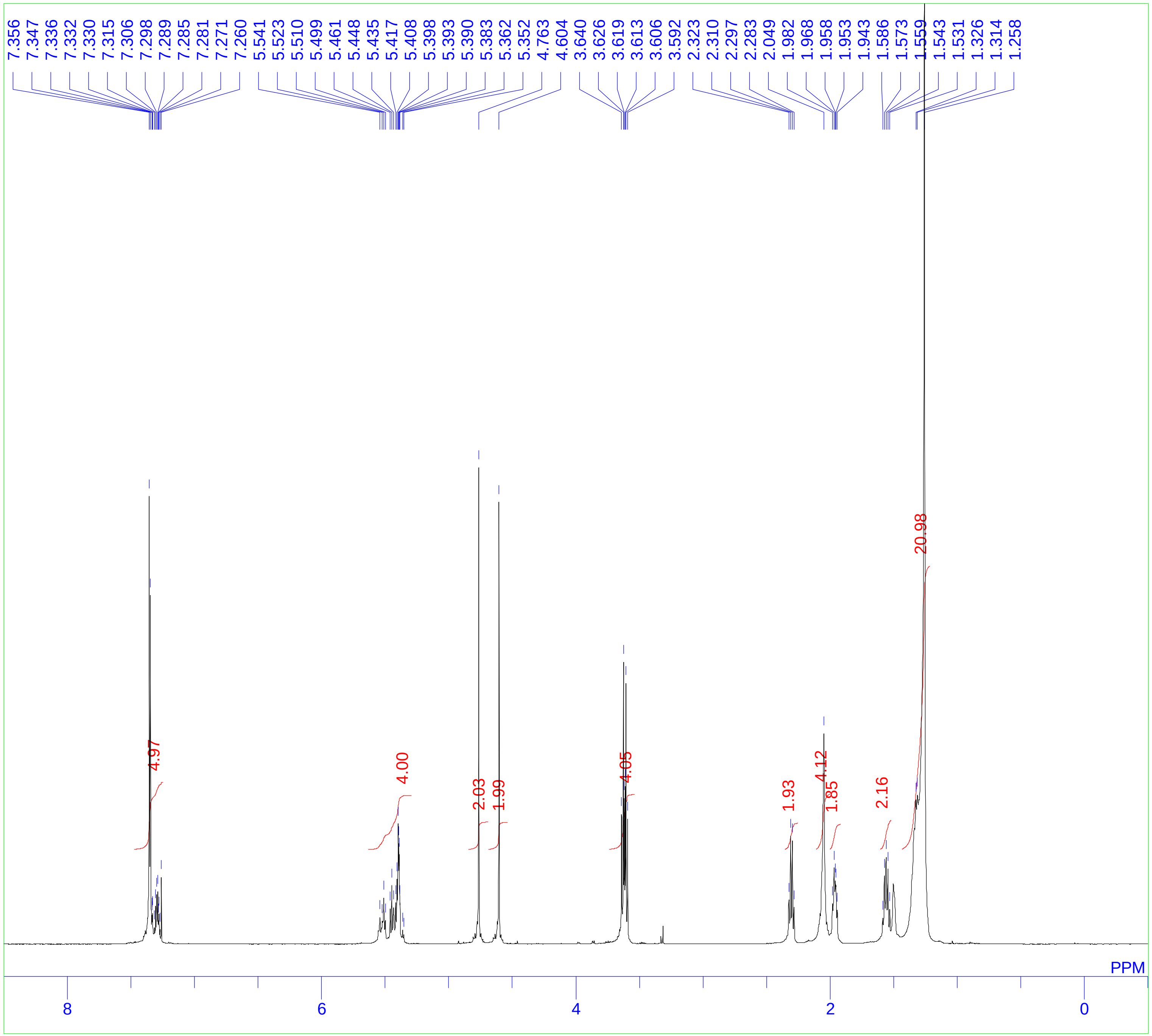

DFILE	IT-3-006re_Proton-1-2.als
COMNT	single_pulse
DATIM	2016-07-29 19:33:07
OBNUC	1H
EXMOD	proton.jxp
OBFRQ	500.16 MHz
OBSET	2.41 KHz
OBFIN	6.01 Hz
POINT	16384
FREQU	9384.38 Hz
SCANS	8
ACQTM	1.7459 sec
PD	5.0000 sec
PW1	3.52 usec
IRNUC	1H
CTEMP	23.1 c
SLVNT	CDCL3
EXREF	7.26 ppm
BF	0.25 Hz
RGAIN	22

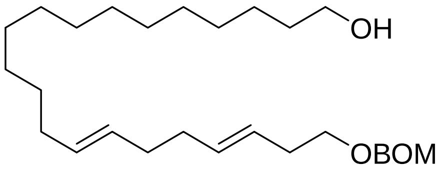

(14*E*,18*E*)-21-Benzyloxymethoxyhenicosa-14,18-dien-1-ol (**10**)

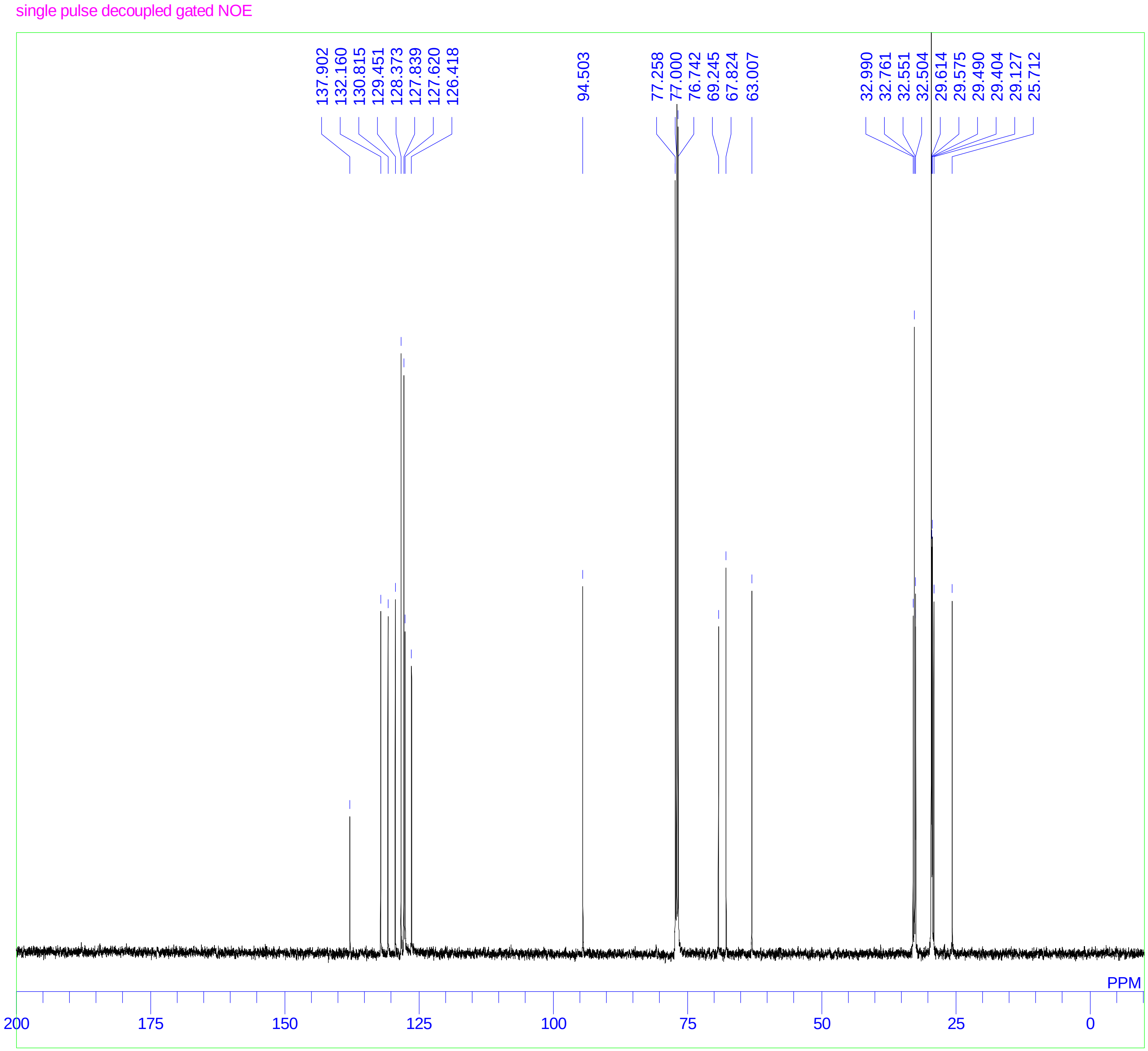

DFILE	IT-3-006re_Carbon-1-2.als
COMNT	single pulse decoupled gated NOE
DATIM	2016-07-29 19:42:55
OBNUC	13C
EXMOD	carbon.jxp
OBFRQ	125.77 MHz
OBSET	7.87 KHz
OBFIN	4.21 Hz
POINT	32768
FREQU	39308.18 Hz
SCANS	530
ACQTM	0.8336 sec
PD	2.0000 sec
PW1	3.74 usec
IRNUC	1H
CTEMP	23.1 c
SLVNT	CDCL3
EXREF	77.00 ppm
BF	0.25 Hz
RGAIN	24

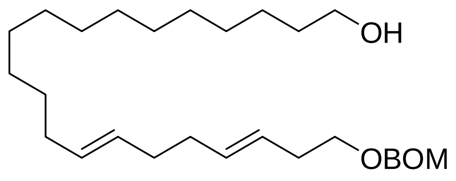

(14*E*,18*E*)-21-Benzyloxymethoxyhenicosa-14,18-dien-1-ol (**10**)

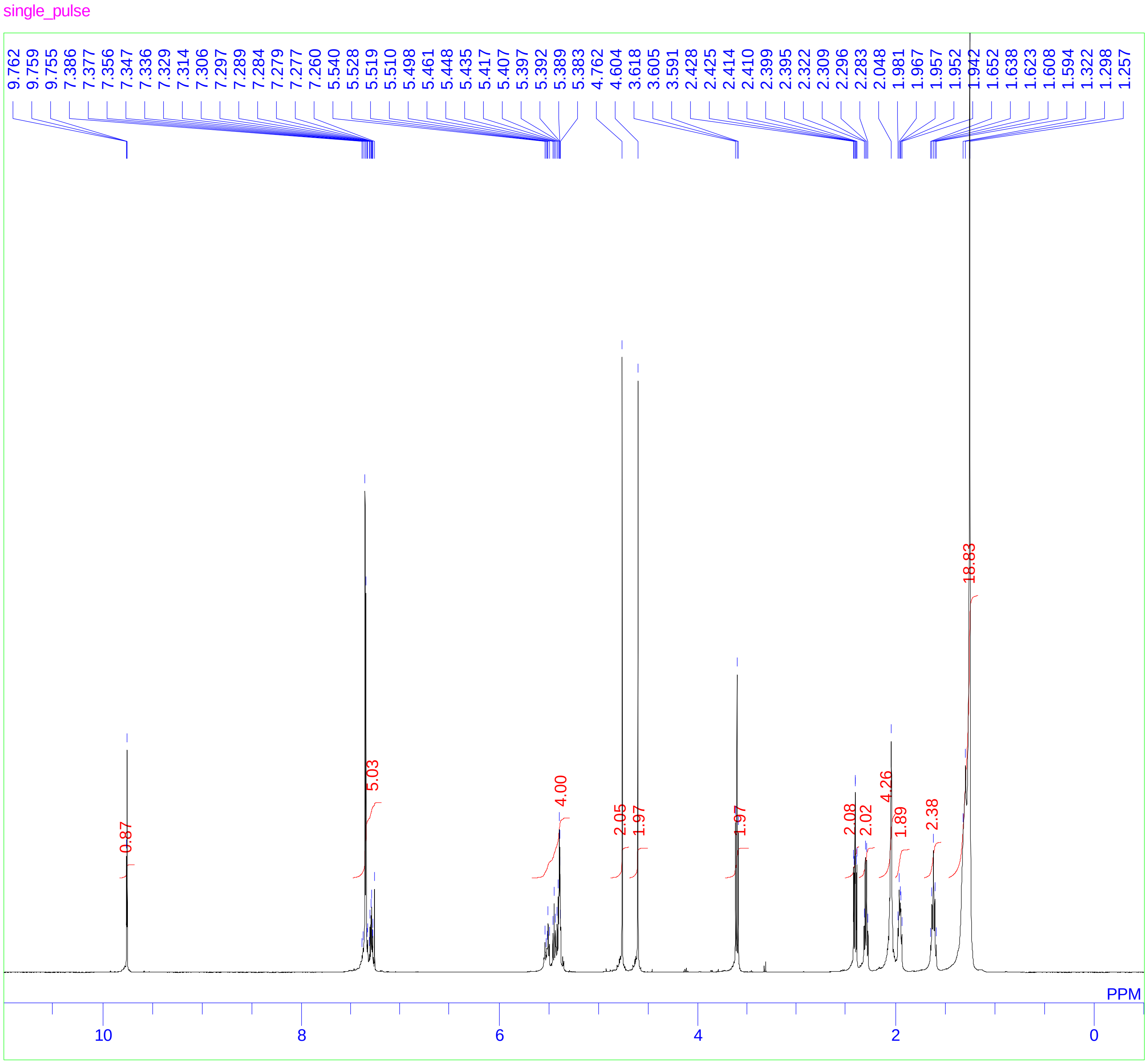

DFILE	IT-3-033_Proton-1-2.als
COMNT	single_pulse
DATIM	2016-09-06 18:35:41
OBNUC	1H
EXMOD	proton.jxp
OBFRQ	500.16 MHz
OBSET	2.41 KHz
OBFIN	6.01 Hz
POINT	16384
FREQU	9384.38 Hz
SCANS	8
ACQTM	1.7459 sec
PD	5.0000 sec
PW1	3.52 usec
IRNUC	1H
CTEMP	23.4 c
SLVNT	CDCL3
EXREF	7.26 ppm
BF	0.25 Hz
RGAIN	24

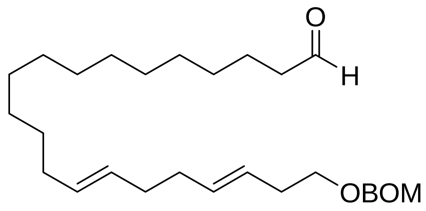

(14*E*,18*E*)-21-Benzyloxymethoxyhenicosa-14,18-dienal (**6**)

single pulse decoupled gated NOE

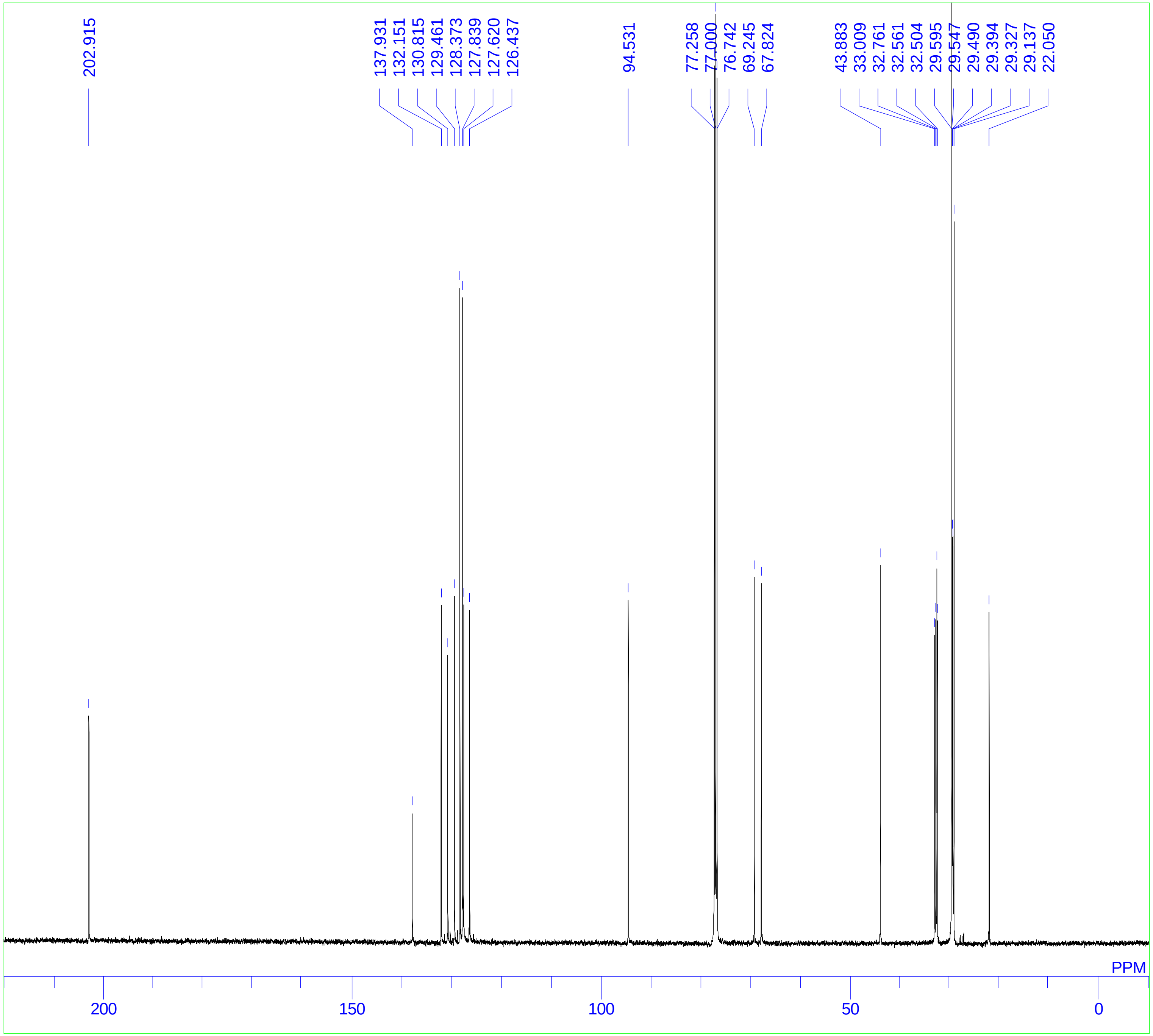

DFILE	IT-3-033_Carbon-1-2.als
COMNT	single pulse decoupled gated NOE
DATIM	2016-09-07 01:16:25
OBNUC	13C
EXMOD	carbon.jxp
OBFRQ	125.77 MHz
OBSET	7.87 KHz
OBFIN	4.21 Hz
POINT	32768
FREQU	39308.18 Hz
SCANS	4000
ACQTM	0.8336 sec
PD	2.0000 sec
PW1	3.74 usec
IRNUC	1H
CTEMP	23.5 c
SLVNT	CDCL3
EXREF	77.00 ppm
BF	0.25 Hz
RGAIN	24

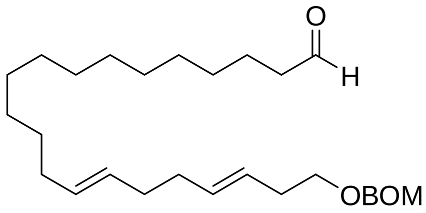

(14*E*,18*E*)-21-Benzyloxymethoxyhenicosa-14,18-dienal (**6**)

single_pulse

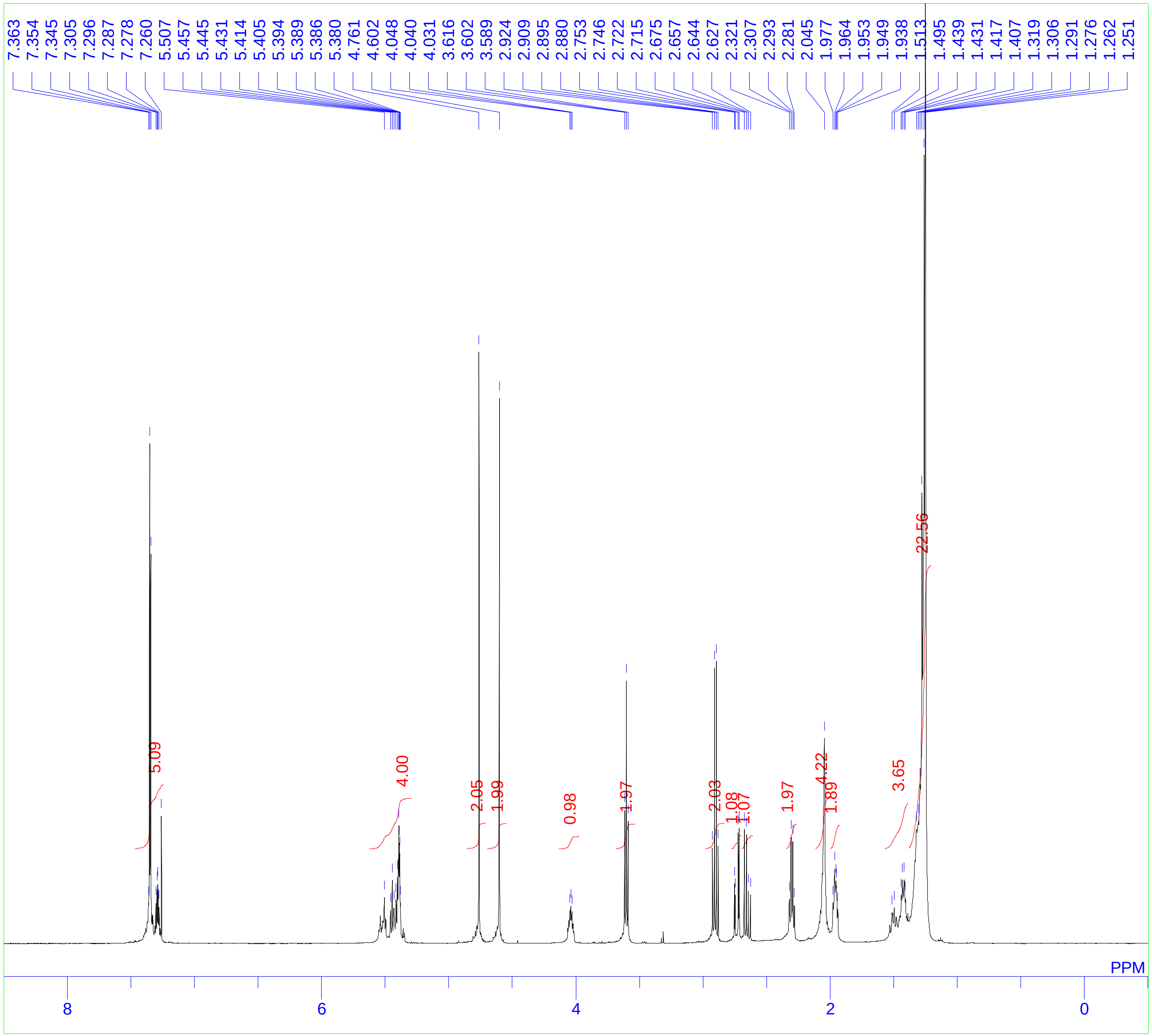

DFILE	IT-3-035_Proton-1-2.als
COMNT	single_pulse
DATIM	2016-09-08 14:43:40
OBNUC	1H
EXMOD	proton.jxp
OBFRQ	500.16 MHz
OBSET	2.41 KHz
OBFIN	6.01 Hz
POINT	16384
FREQU	9384.38 Hz
SCANS	8
ACQTM	1.7459 sec
PD	5.0000 sec
PW1	3.52 usec
IRNUC	1H
CTEMP	23.4 c
SLVNT	CDCL3
EXREF	7.26 ppm
BF	0.25 Hz
RGAIN	26

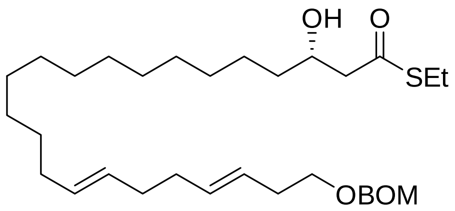

(3*S*,16*E*,20*E*)-Ethyl 23-benzyloxymethoxy-3-hydroxytricosa-16,20-dienethioate (**11**)

single pulse decoupled gated NOE

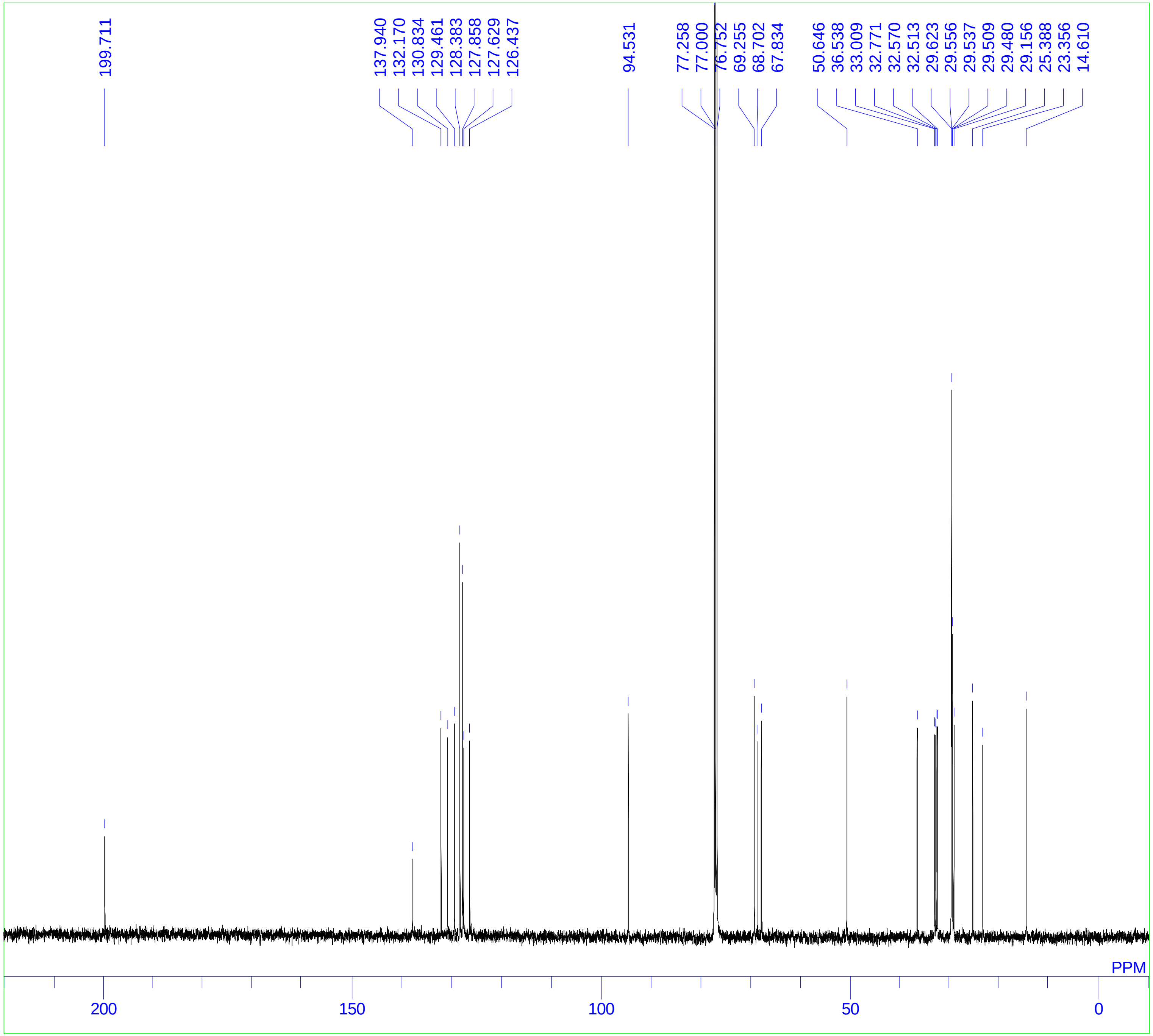

DFILE	IT-3-035_Carbon-1-2.als
COMNT	single pulse decoupled gated NOE
DATIM	2016-09-08 17:59:20
OBNUC	13C
EXMOD	carbon.jxp
OBFRQ	125.77 MHz
OBSET	7.87 KHz
OBFIN	4.21 Hz
POINT	32768
FREQU	39308.18 Hz
SCANS	535
ACQTM	0.8336 sec
PD	2.0000 sec
PW1	3.74 usec
IRNUC	1H
CTEMP	23.4 c
SLVNT	CDCL3
EXREF	77.00 ppm
BF	0.25 Hz
RGAIN	24

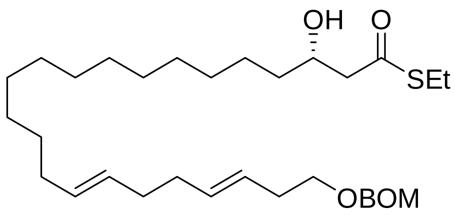

(3*S*,16*E*,20*E*)-Ethyl 23-benzyloxymethoxy-3-hydroxytricosa-16,20-dienethioate (**11**)

single_pulse

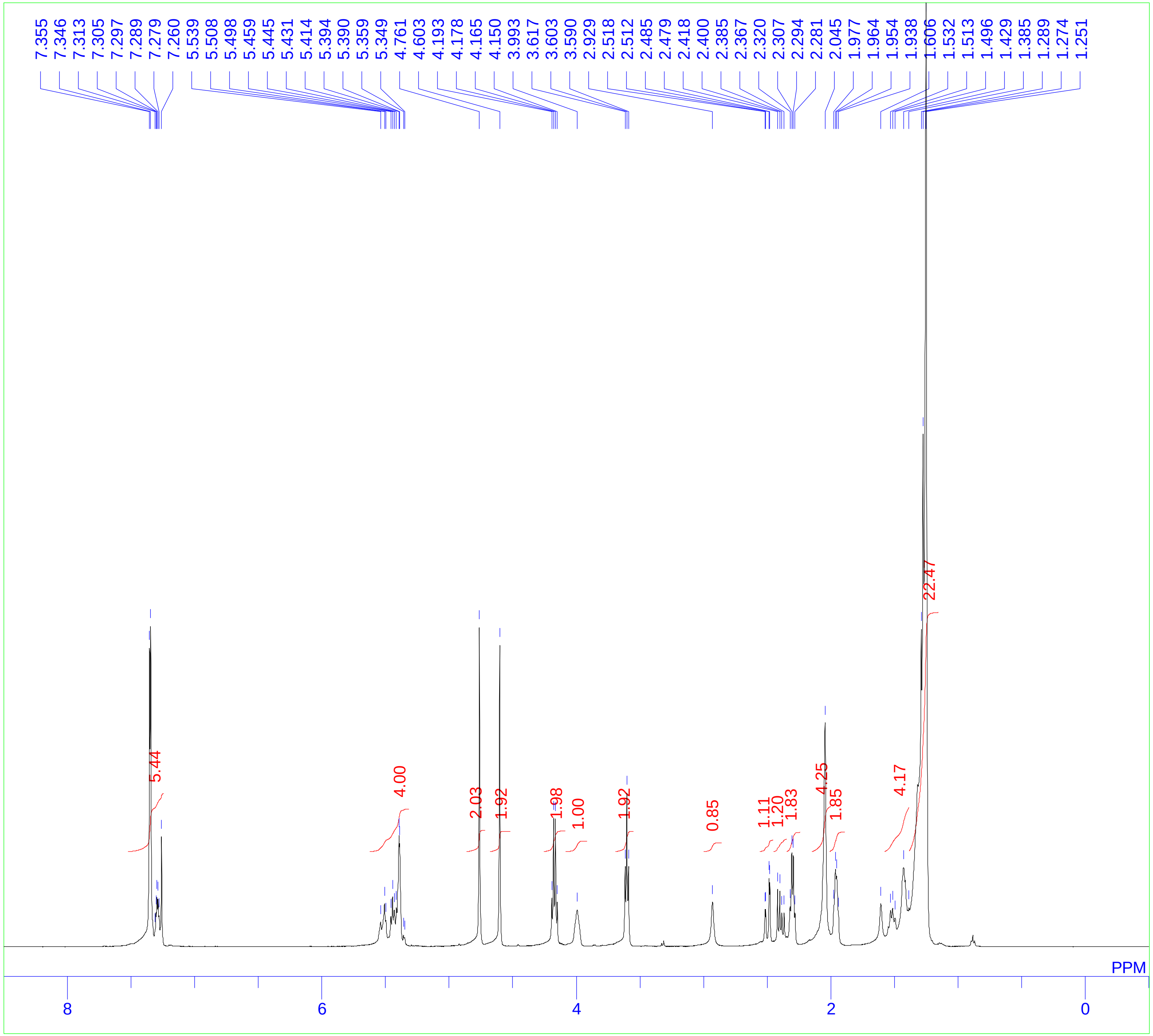

DFILE	IT-3-040_Proton-1-2.als
COMNT	single_pulse
DATIM	2016-09-13 11:06:38
OBNUC	1H
EXMOD	proton.jxp
OBFRQ	500.16 MHz
OBSET	2.41 KHz
OBFIN	6.01 Hz
POINT	16384
FREQU	9384.38 Hz
SCANS	8
ACQTM	1.7459 sec
PD	5.0000 sec
PW1	3.52 usec
IRNUC	1H
CTEMP	23.2 c
SLVNT	CDCL3
EXREF	7.26 ppm
BF	0.25 Hz
RGAIN	30

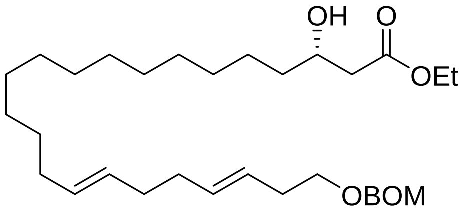

(3*S*,16*E*,20*E*)-Ethyl 23-benzyloxymethoxy-3-hydroxytricosa-16,20-dienoate (**12**)

single pulse decoupled gated NOE

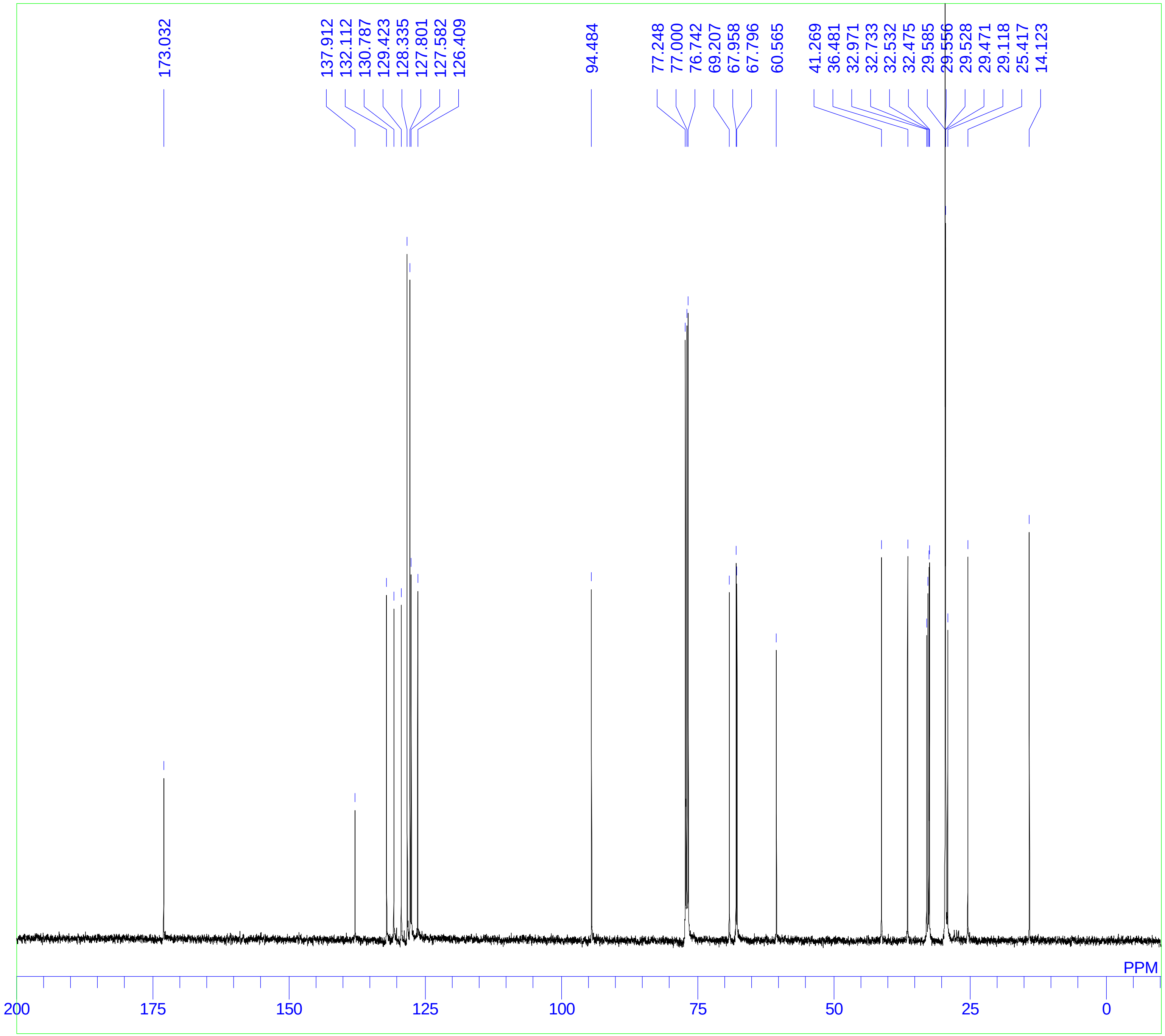

DFILE	IT-3-040_Carbon-1-2.als
COMNT	single pulse decoupled gated NOE
DATIM	2016-09-17 17:37:57
OBNUC	13C
EXMOD	carbon.jxp
OBFRQ	125.77 MHz
OBSET	7.87 KHz
OBFIN	4.21 Hz
POINT	32768
FREQU	39308.18 Hz
SCANS	542
ACQTM	0.8336 sec
PD	2.0000 sec
PW1	3.74 usec
IRNUC	1H
CTEMP	24.5 c
SLVNT	CDCL3
EXREF	77.00 ppm
BF	0.25 Hz
RGAIN	26

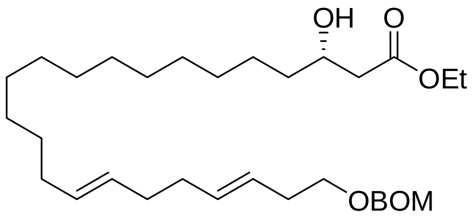

(3*S*,16*E*,20*E*)-Ethyl 23-benzyloxymethoxy-3-hydroxytricosa-16,20-dienoate (**12**)

single_pulse

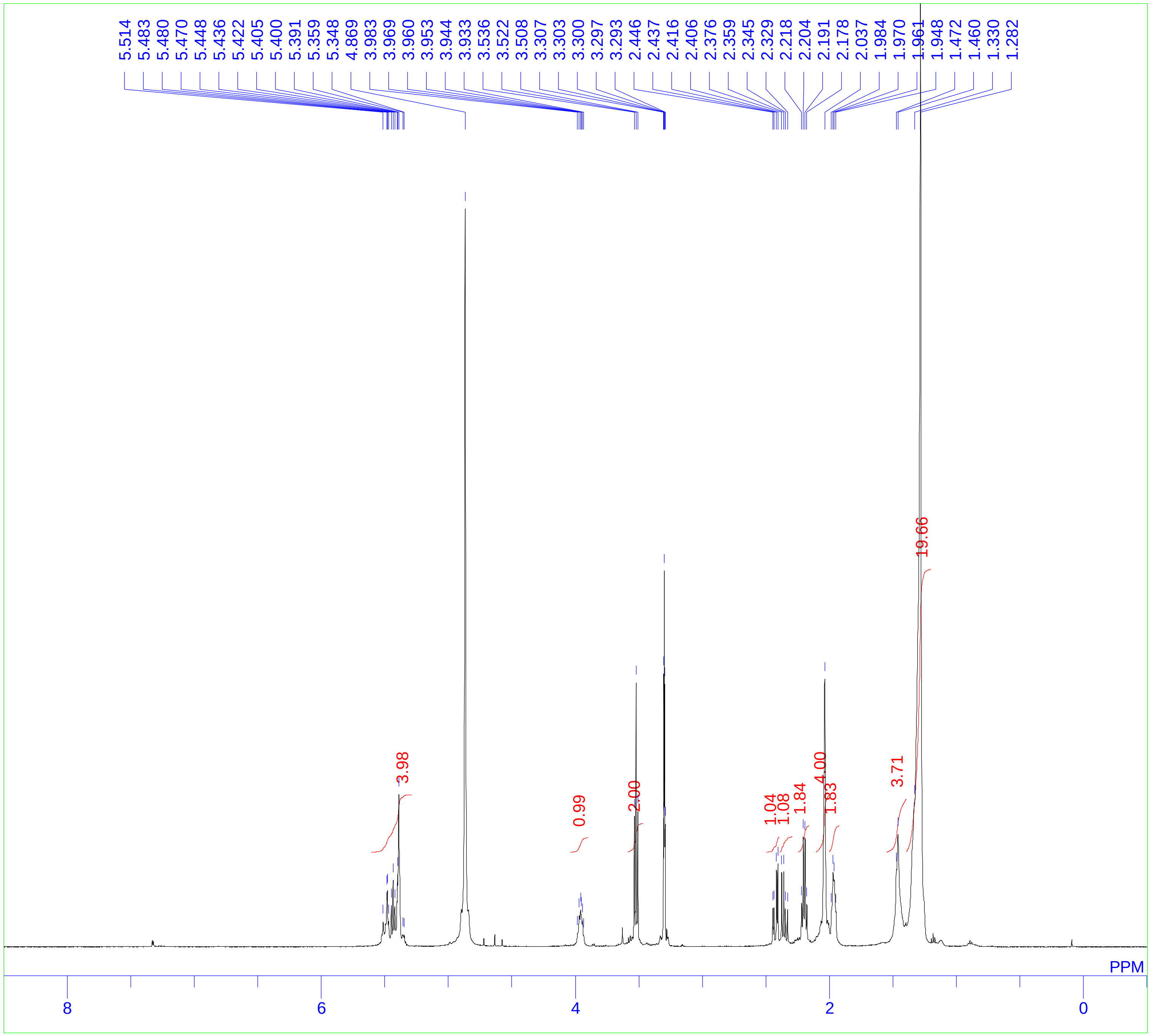

DFILE	IT-3-062_Proton-1-2.als
COMNT	single_pulse
DATIM	2016-09-30 16:47:28
OBNUC	1H
EXMOD	proton.jxp
OBFRQ	500.16 MHz
OBSET	2.41 KHz
OBFIN	6.01 Hz
POINT	16384
FREQU	9384.38 Hz
SCANS	8
ACQTM	1.7459 sec
PD	5.0000 sec
PW1	3.52 usec
IRNUC	1H
CTEMP	23.3 c
SLVNT	CD3OD
EXREF	3.30 ppm
BF	0.25 Hz
RGAIN	42

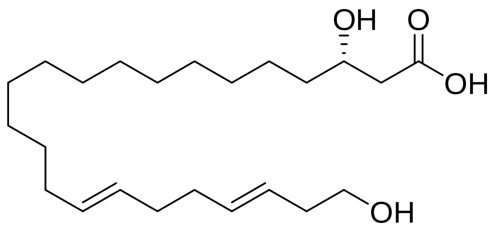

(3*S*,16*E*,20*E*)-3,23-Dihydroxytricosa-16,20-dienoic acid (**5**)

single pulse decoupled gated NOE

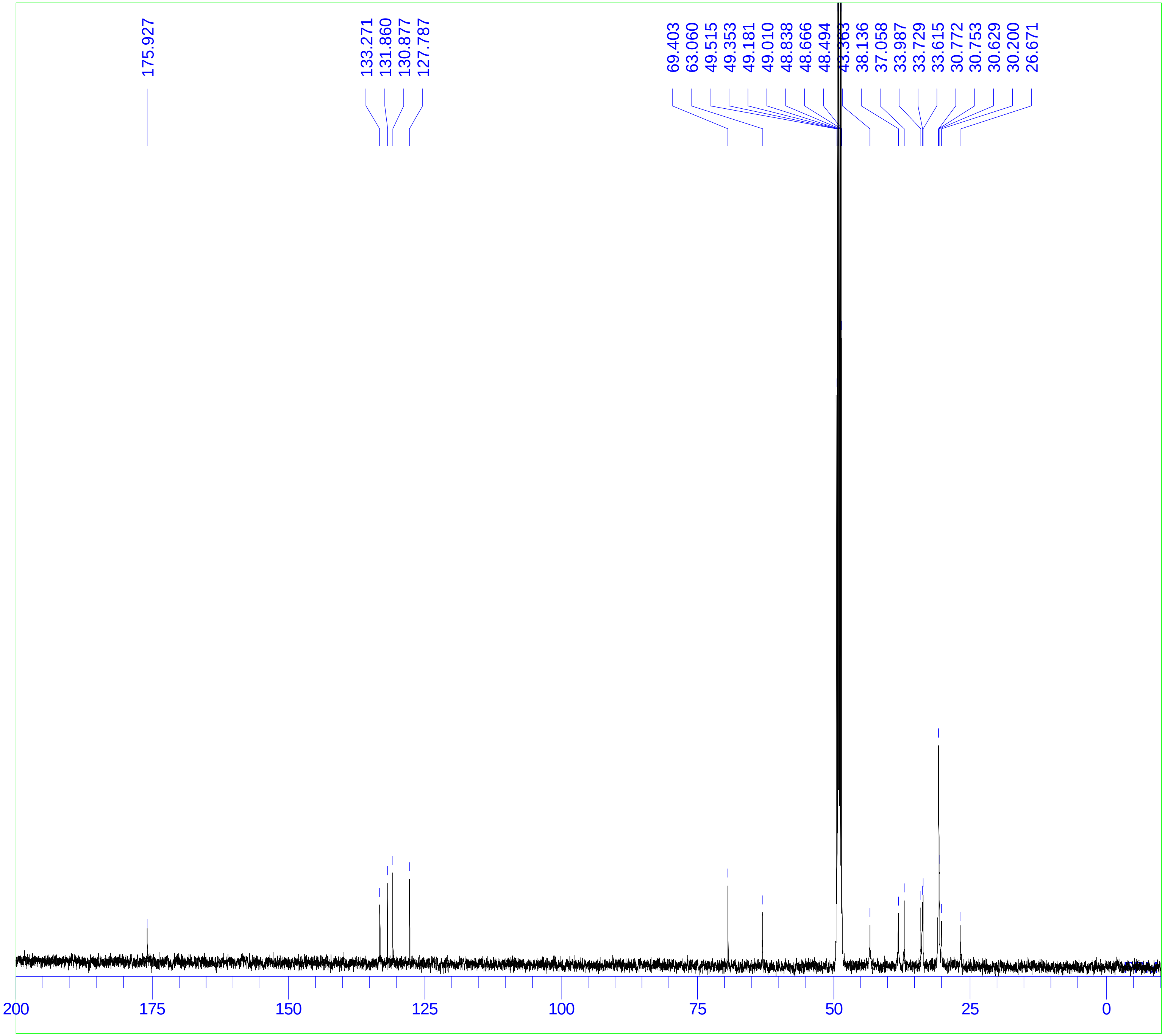

DFILE	IT-3-062_Carbon-1-2.als
COMNT	single pulse decoupled gated NOE
DATIM	2016-09-30 16:49:16
OBNUC	13C
EXMOD	carbon.jxp
OBFRQ	125.77 MHz
OBSET	7.87 KHz
OBFIN	4.21 Hz
POINT	32768
FREQU	39308.18 Hz
SCANS	742
ACQTM	0.8336 sec
PD	2.0000 sec
PW1	3.74 usec
IRNUC	1H
CTEMP	23.2 c
SLVNT	CD3OD
EXREF	49.00 ppm
BF	0.25 Hz
RGAIN	22

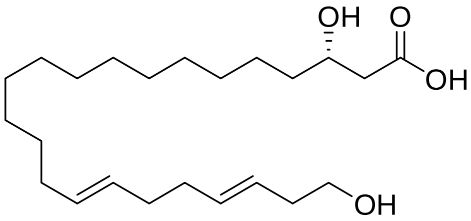

(3*S*,16*E*,20*E*)-3,23-Dihydroxytricosa-16,20-dienoic acid (**5**)

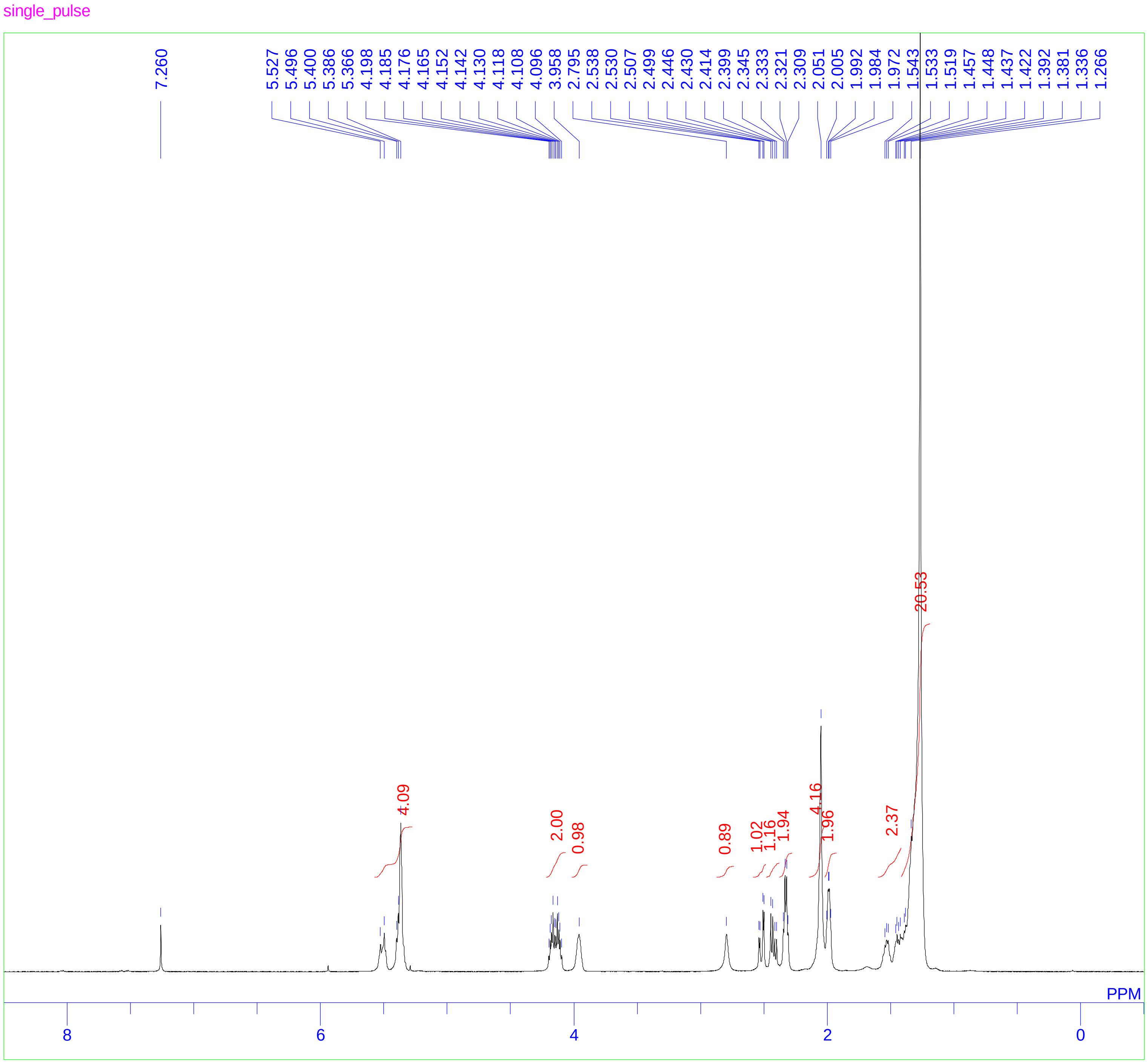

DFILE	IT-3-064_Proton-1-2.als
COMNT	single_pulse
DATIM	2016-10-01 14:25:22
OBNUC	1H
EXMOD	proton.jxp
OBFRQ	500.16 MHz
OBSET	2.41 KHz
OBFIN	6.01 Hz
POINT	16384
FREQU	9384.38 Hz
SCANS	8
ACQTM	1.7459 sec
PD	5.0000 sec
PW1	3.52 usec
IRNUC	1H
CTEMP	23.0 c
SLVNT	CDCL3
EXREF	7.26 ppm
BF	0.25 Hz
RGAIN	46

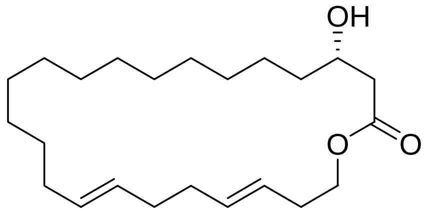

(3*S*,16*E*,20*E*)-3-Hydroxytricosa-16,20-dienolide (**4**)

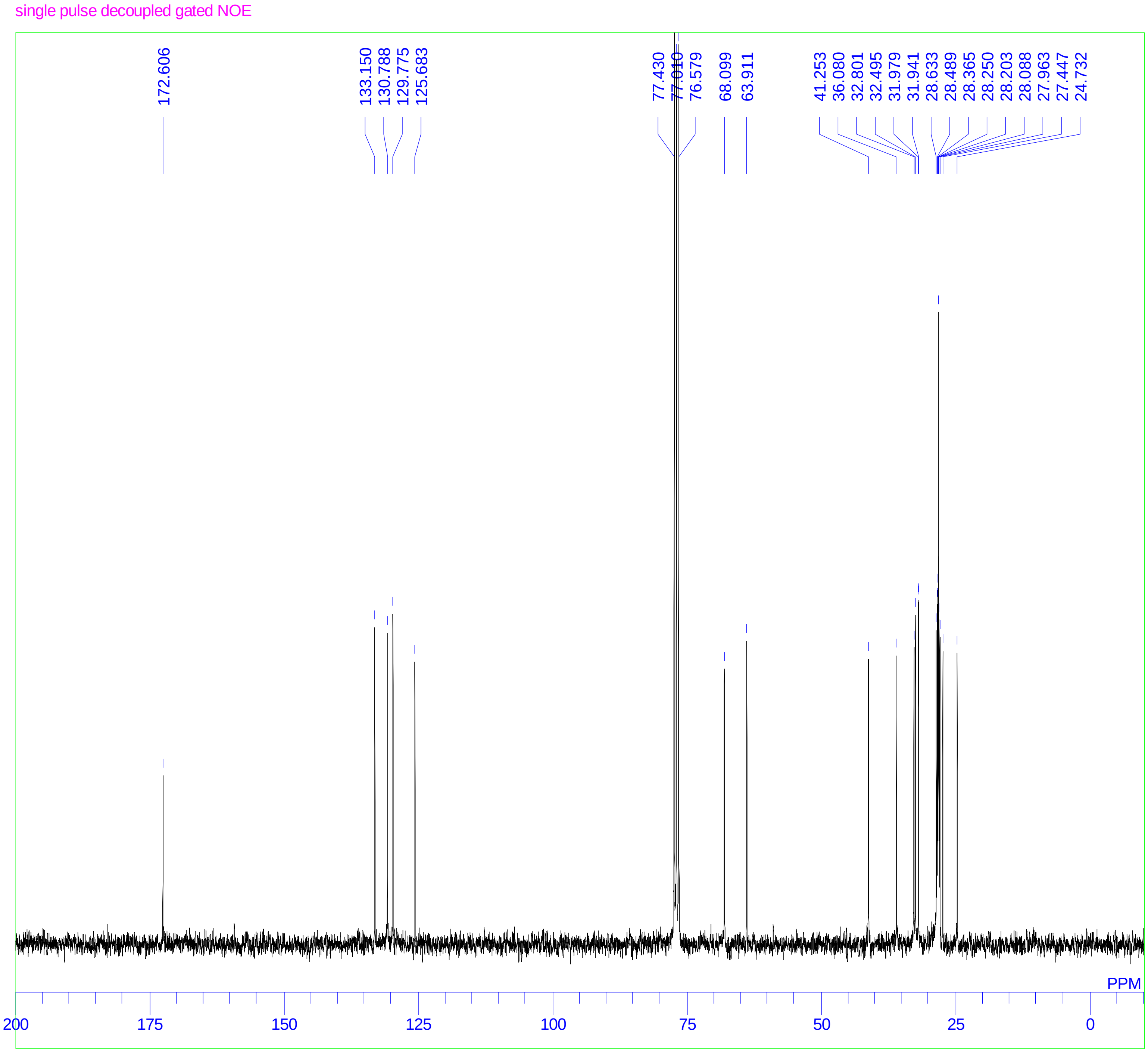

DFILE	IT-3-064_Carbon-1-2.als
COMNT	single pulse decoupled gated NOE
DATIM	2016-10-06 17:30:09
OBNUC	13C
EXMOD	carbon.jxp
OBFRQ	75.57 MHz
OBSET	5.79 KHz
OBFIN	1.08 Hz
POINT	32768
FREQU	23674.24 Hz
SCANS	406
ACQTM	1.3841 sec
PD	2.0000 sec
PW1	3.27 usec
IRNUC	1H
CTEMP	22.8 c
SLVNT	CDCL3
EXREF	77.00 ppm
BF	0.25 Hz
RGAIN	50

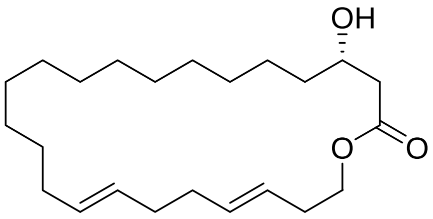

(3*S*,16*E*,20*E*)-3-Hydroxytricosa-16,20-dienolide (**4**)

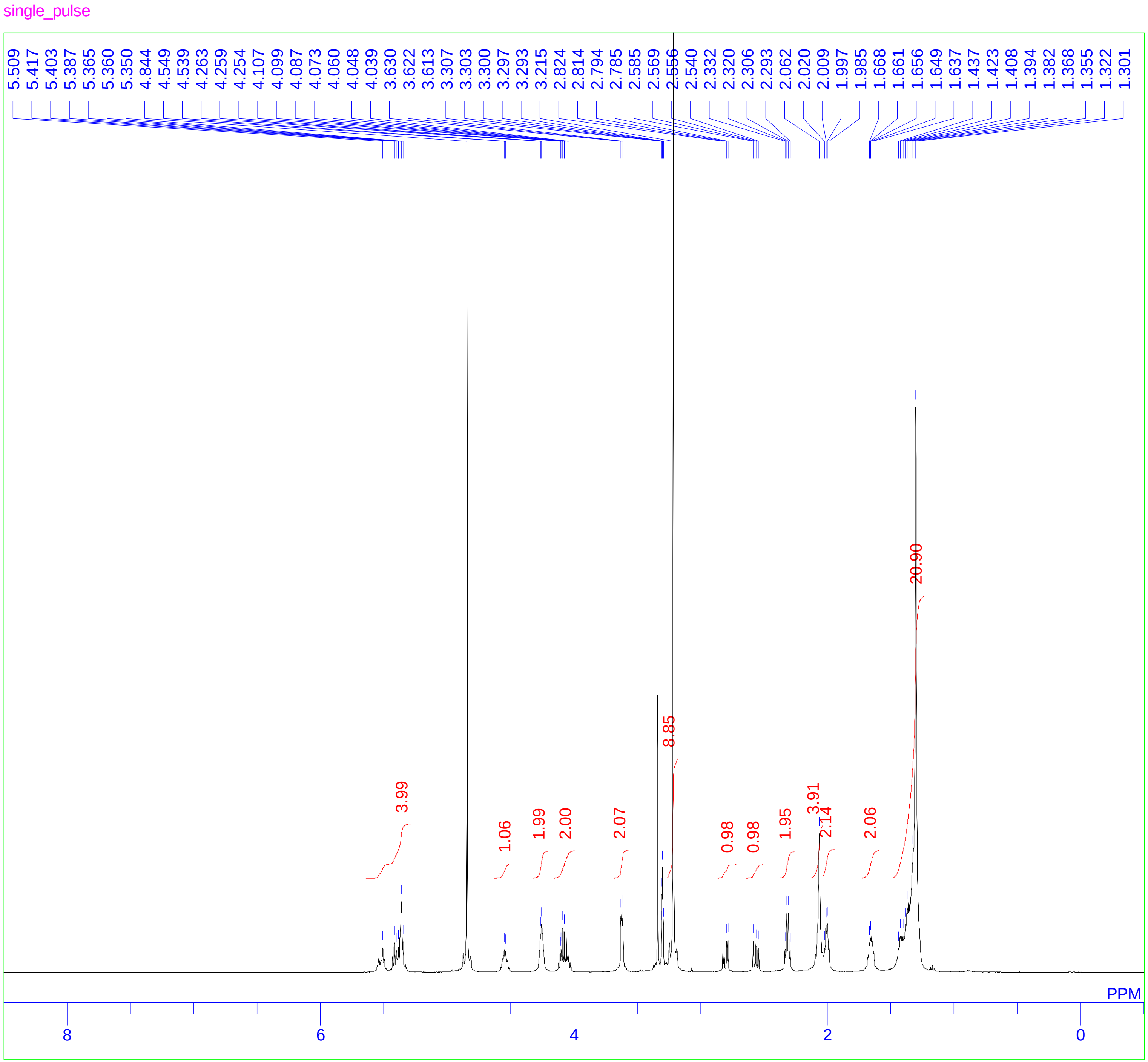

DFILE	IT-3-066_Proton-2-2.als
COMNT	single_pulse
DATIM	2016-10-12 22:12:21
OBNUC	1H
EXMOD	proton.jxp
OBFRQ	500.16 MHz
OBSET	2.41 KHz
OBFIN	6.01 Hz
POINT	16384
FREQU	9384.38 Hz
SCANS	16
ACQTM	1.7459 sec
PD	5.0000 sec
PW1	3.52 usec
IRNUC	1H
CTEMP	25.0 c
SLVNT	CD3OD
EXREF	3.30 ppm
BF	0.25 Hz
RGAIN	30

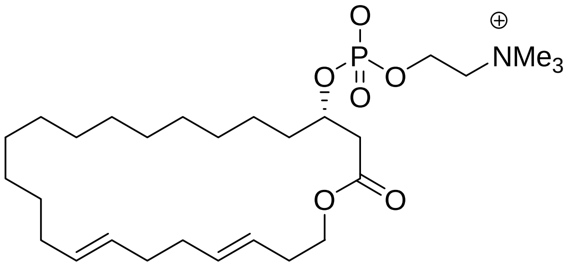

23-Demethyleushearilide (**3**)

single pulse decoupled gated NOE

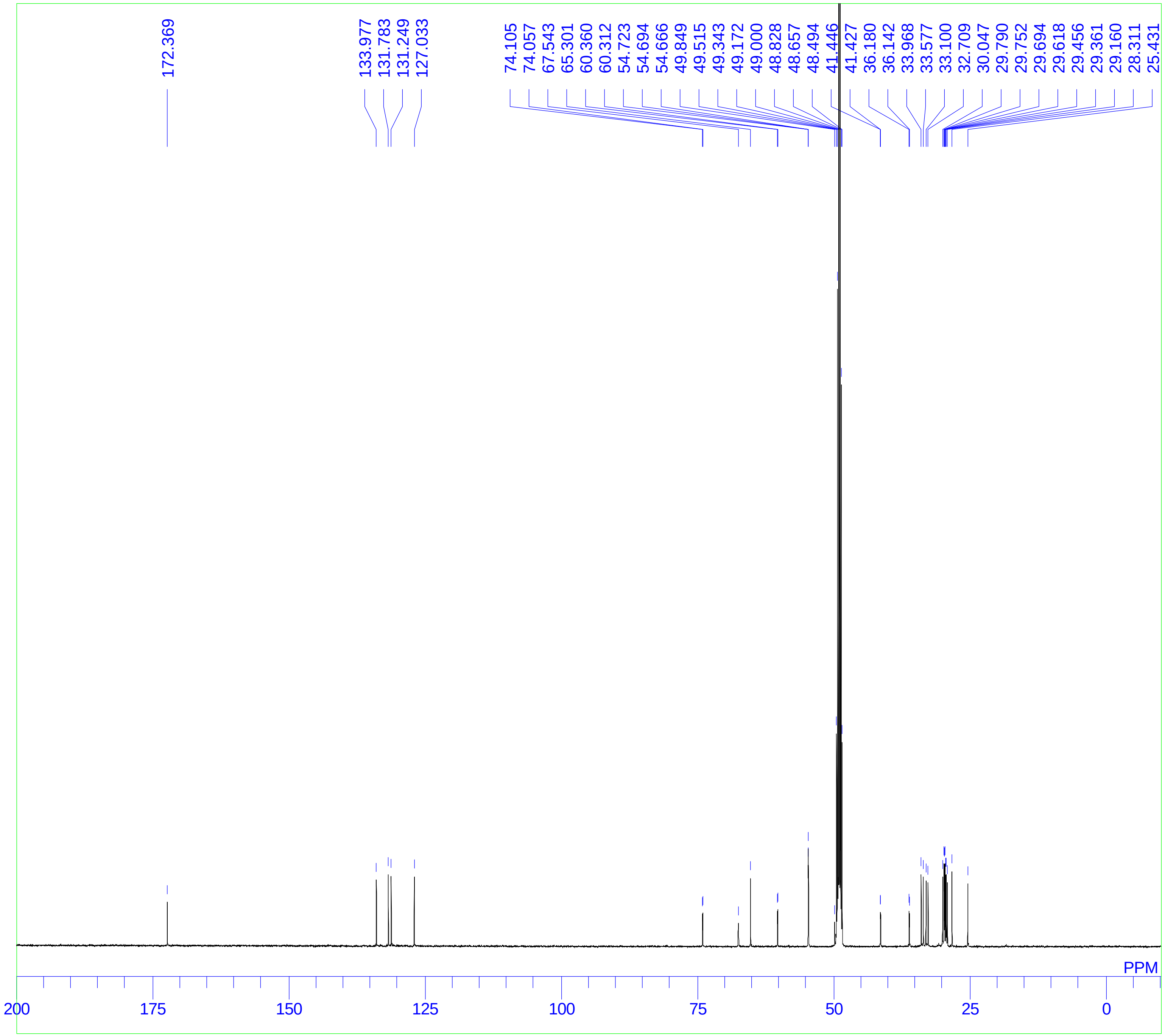

DFILE	IT-3-066_Carbon-1-2.als
COMNT	single pulse decoupled gated NOE
DATIM	2016-10-13 04:06:44
OBNUC	13C
EXMOD	carbon.jxp
OBFRQ	125.77 MHz
OBSET	7.87 KHz
OBFIN	4.21 Hz
POINT	32768
FREQU	39308.18 Hz
SCANS	6014
ACQTM	0.8336 sec
PD	2.0000 sec
PW1	3.74 usec
IRNUC	1H
CTEMP	25.0 c
SLVNT	CD3OD
EXREF	49.00 ppm
BF	0.25 Hz
RGAIN	26

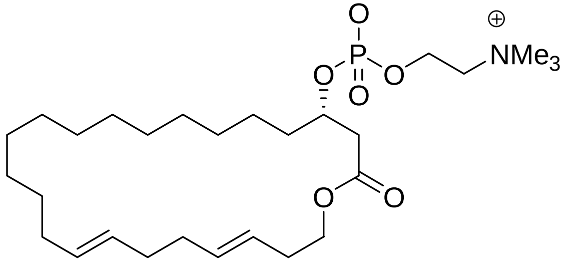

23-Demethyleushearilide (**3**)

single_pulse

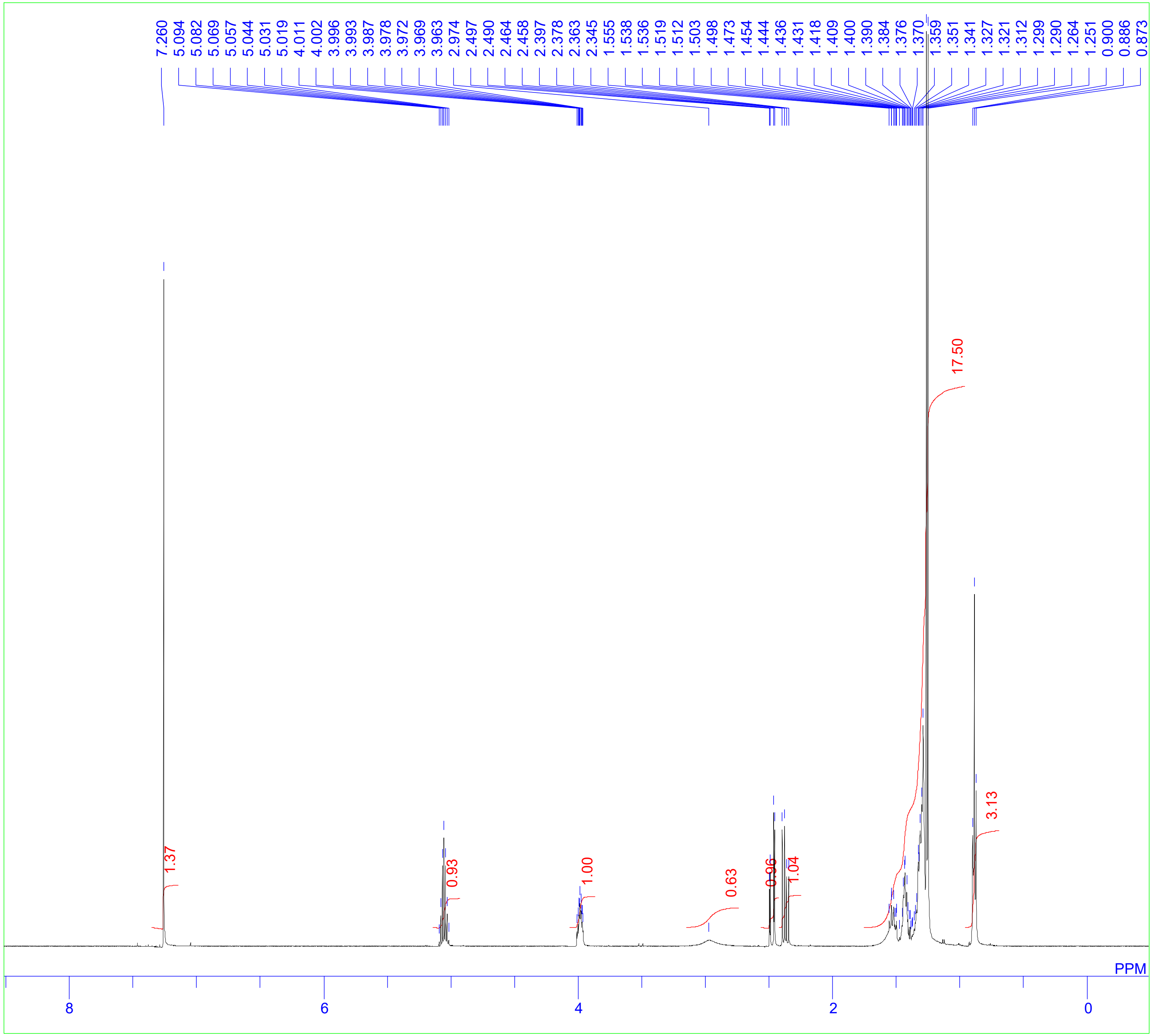

DFILE MA-1-139-Aldol__Proton-1-4.als
COMNT single_pulse
DATIM 2019-06-03 20:24:17
OBNUC 1H
EXMOD proton.jxp
OBFRQ 500.16 MHz
OBSET 2.41 KHz
OBFIN 6.01 Hz
POINT 13120
FREQU 7507.51 Hz
SCANS 8
ACQTM 1.7459 sec
PD 5.0000 sec
PW1 3.84 usec
IRNUC 1H
CTEMP 24.1 c
SLVNT CDCL3
EXREF 7.26 ppm
BF 0.12 Hz
RGAIN 44

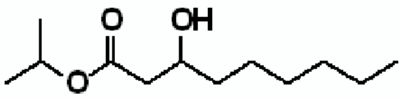

Isopropyl 3-hydroxynonanoate

single pulse decoupled gated NOE

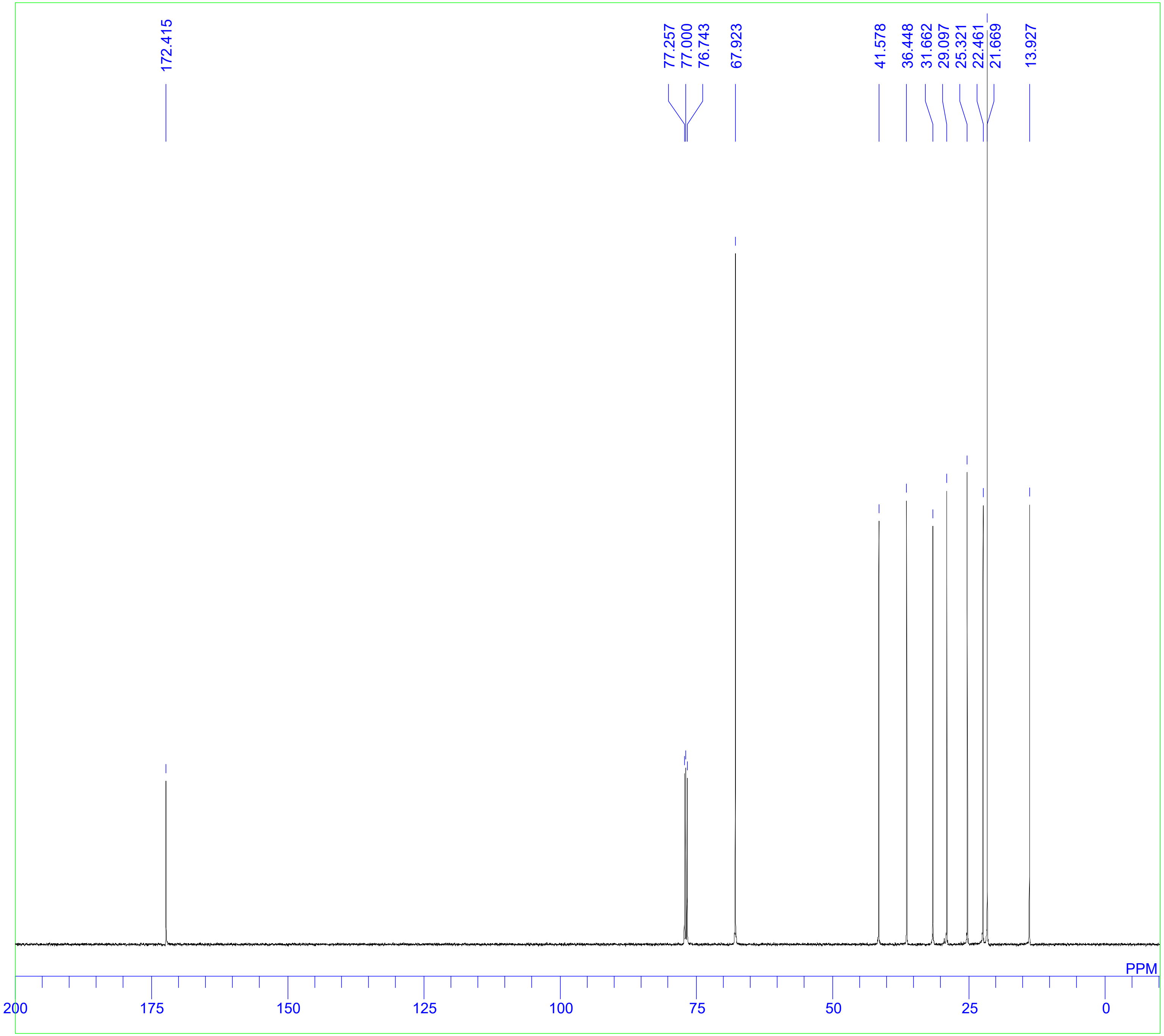

DFILE	ts-1-034-TM_Carbon-2-3.als
COMNT	single pulse decoupled gated NOE
DATIM	2015-09-04 18:16:51
OBNUC	13C
EXMOD	carbon.jxp
OBFRQ	125.77 MHz
OBSET	7.87 KHz
OBFIN	4.21 Hz
POINT	26224
FREQU	31446.54 Hz
SCANS	512
ACQTM	0.8336 sec
PD	2.0000 sec
PW1	3.58 usec
IRNUC	1H
CTEMP	24.3 c
SLVNT	CDCL3
EXREF	77.00 ppm
BF	0.12 Hz
RGAIN	18

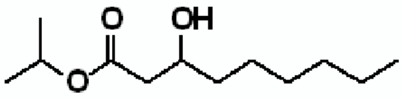

Isopropyl 3-hydroxynonanoate

single_pulse

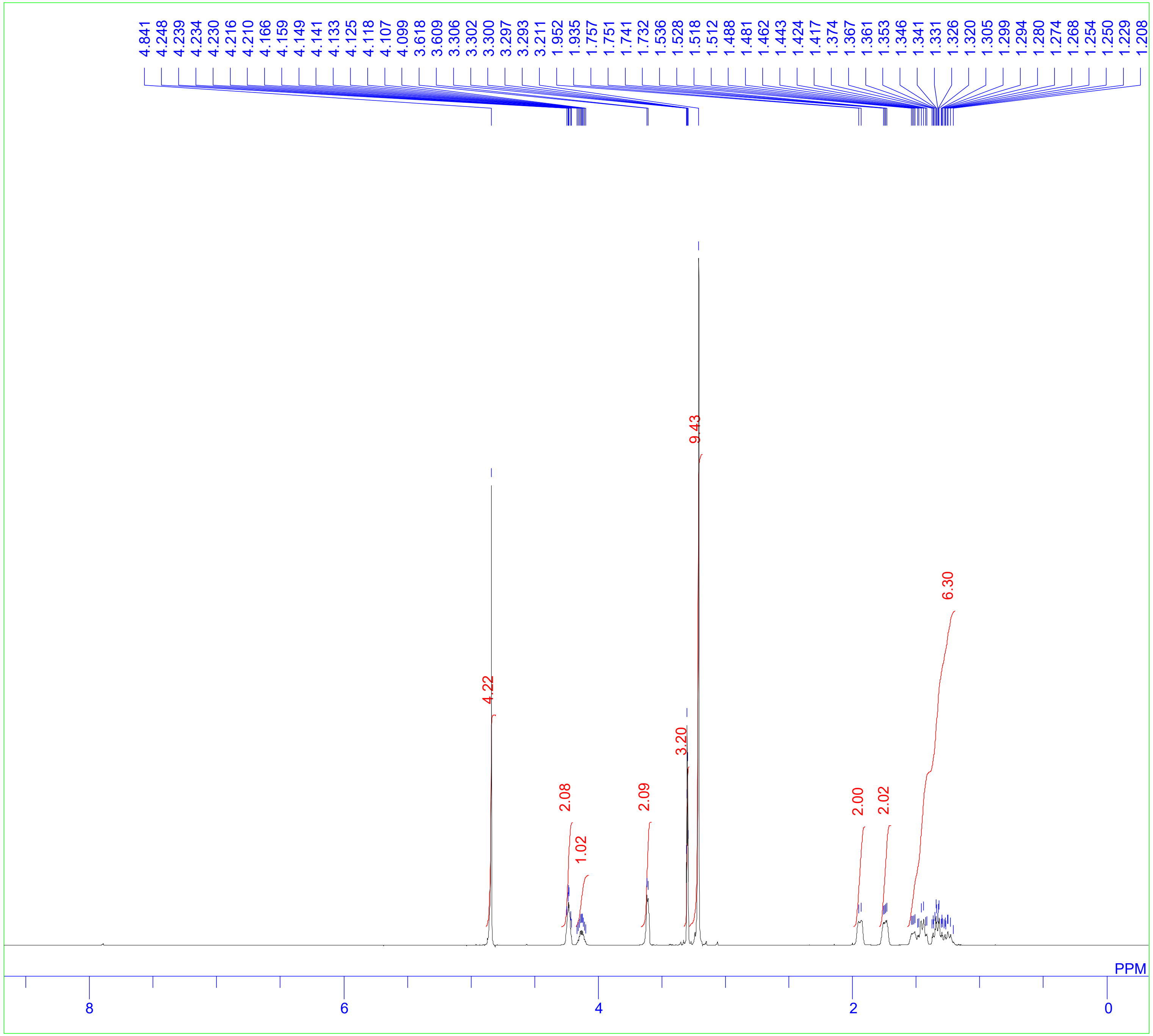

DFILE	YN-28-TM_Proton-1-1.als
COMNT	single_pulse
DATIM	2017-08-01 18:59:30
OBNUC	1H
EXMOD	proton.jxp
OBFRQ	500.16 MHz
OBSET	2.41 KHz
OBFIN	6.01 Hz
POINT	16384
FREQU	9384.38 Hz
SCANS	8
ACQTM	1.7459 sec
PD	5.0000 sec
PW1	3.45 usec
IRNUC	1H
CTEMP	24.6 c
SLVNT	CD3OD
EXREF	3.30 ppm
BF	0.12 Hz
RGAIN	38

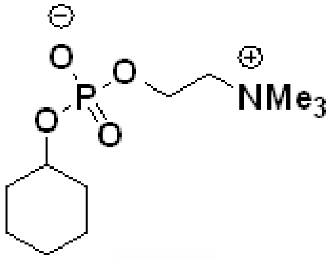

Kderiv

single pulse decoupled gated NOE

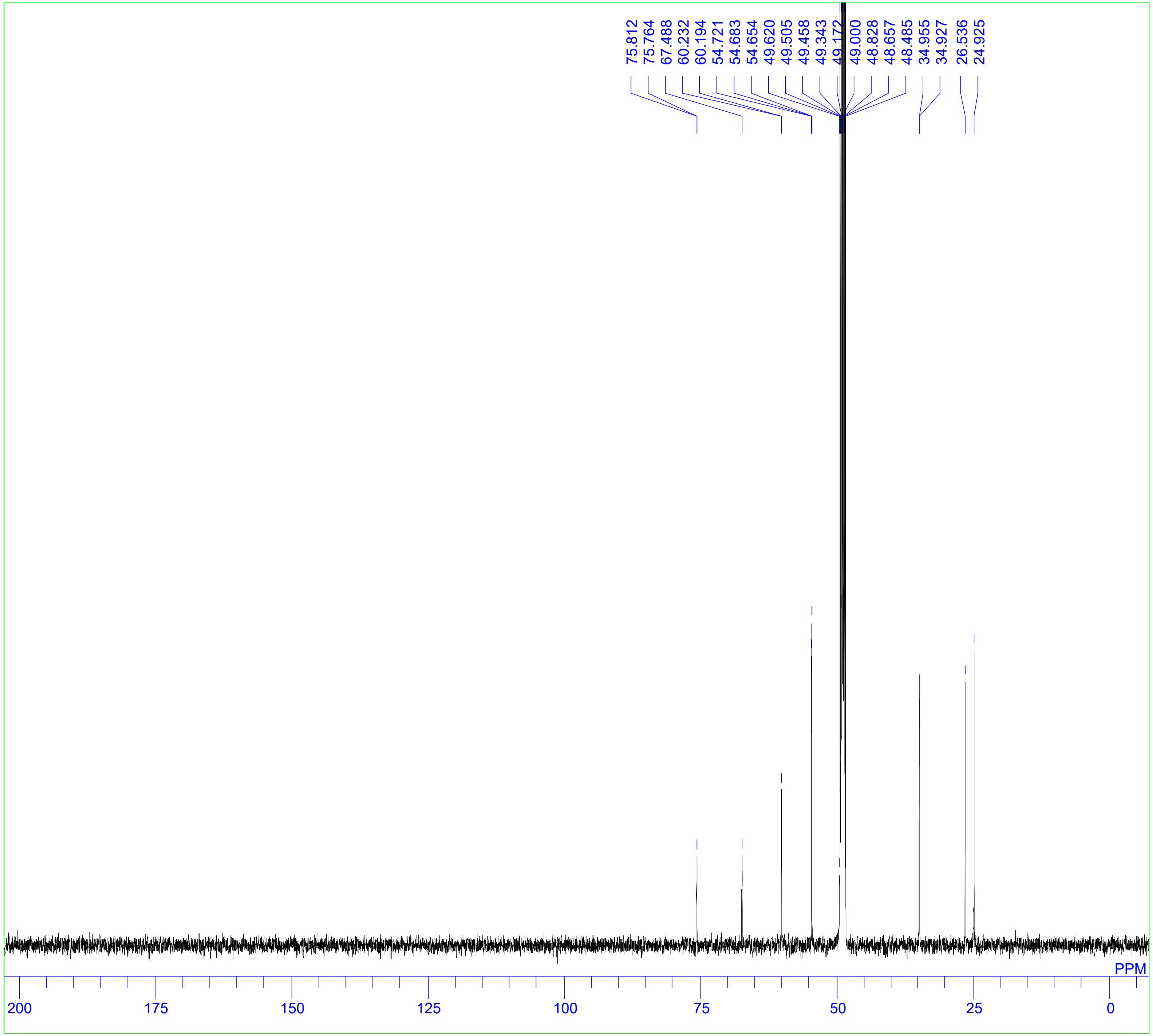

DFILE	MA-1-137-P1_Carbon-1-6.als
COMNT	single pulse decoupled gated NOE
DATIM	2019-05-31 19:10:29
OBNUC	13C
EXMOD	carbon.jxp
OBFRQ	125.77 MHz
OBSET	7.87 KHz
OBFIN	4.21 Hz
POINT	32780
FREQU	39308.18 Hz
SCANS	2048
ACQTM	0.8336 sec
PD	2.0000 sec
PW1	3.87 usec
IRNUC	1H
CTEMP	24.1 c
SLVNT	CD3OD
EXREF	49.00 ppm
BF	0.12 Hz
RGAIN	32

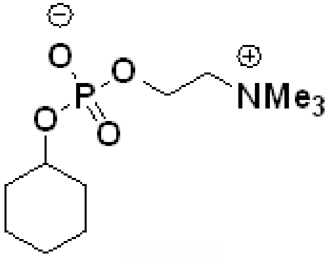

Kderiv

single_pulse

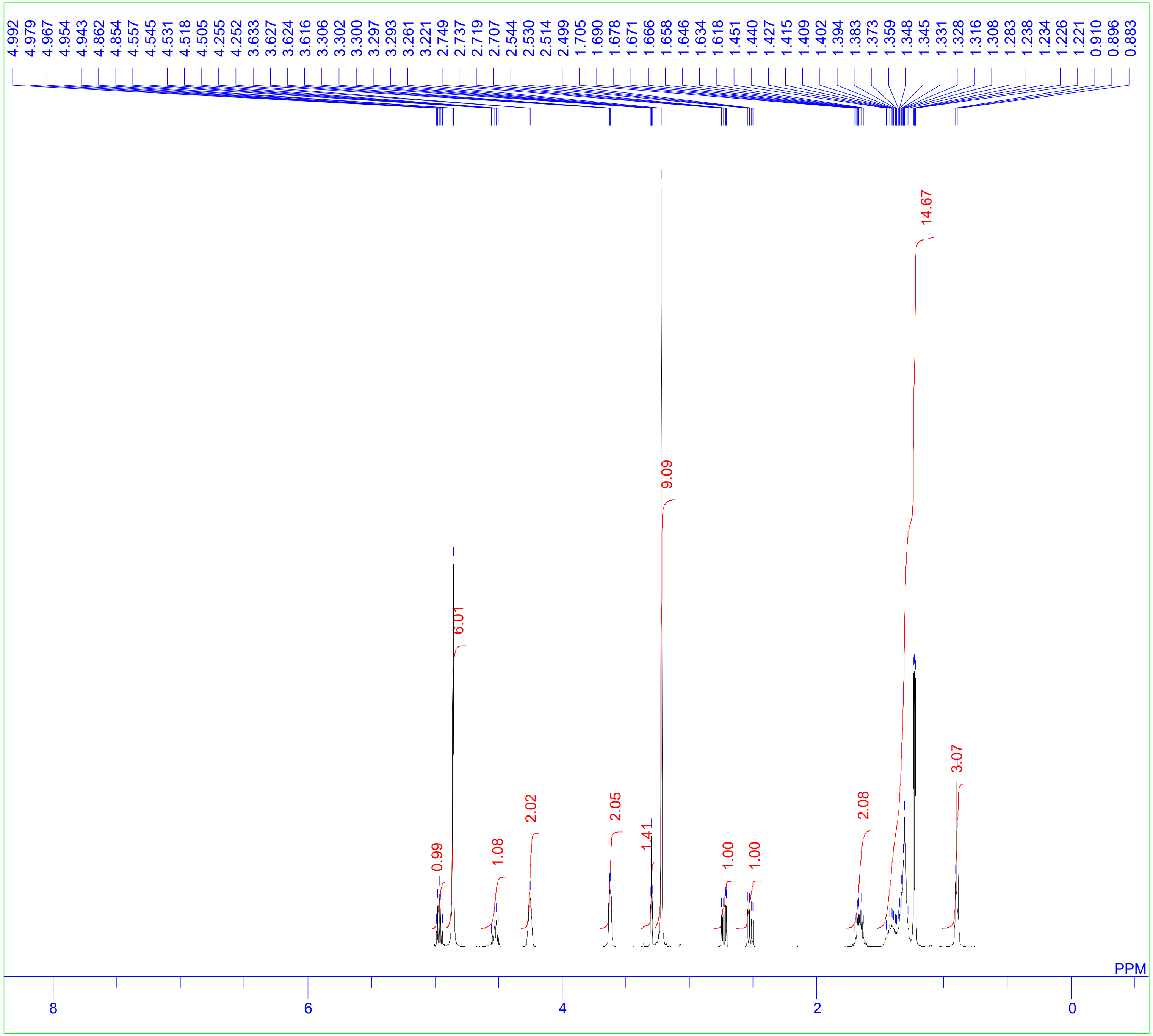

DFILE	YN-24-TM-4_Proton-1-4.als
COMNT	single_pulse
DATIM	2017-07-22 10:08:14
OBNUC	1H
EXMOD	proton.jxp
OBFRQ	500.16 MHz
OBSET	2.41 KHz
OBFIN	6.01 Hz
POINT	13120
FREQU	7507.51 Hz
SCANS	8
ACQTM	1.7459 sec
PD	5.0000 sec
PW1	3.45 usec
IRNUC	1H
CTEMP	23.7 c
SLVNT	CD3OD
EXREF	3.30 ppm
BF	0.12 Hz
RGAIN	28

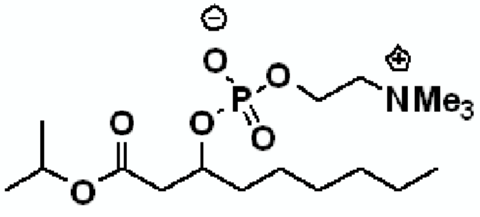

$\mathbf{L}_{deriv}$

single pulse decoupled gated NOE

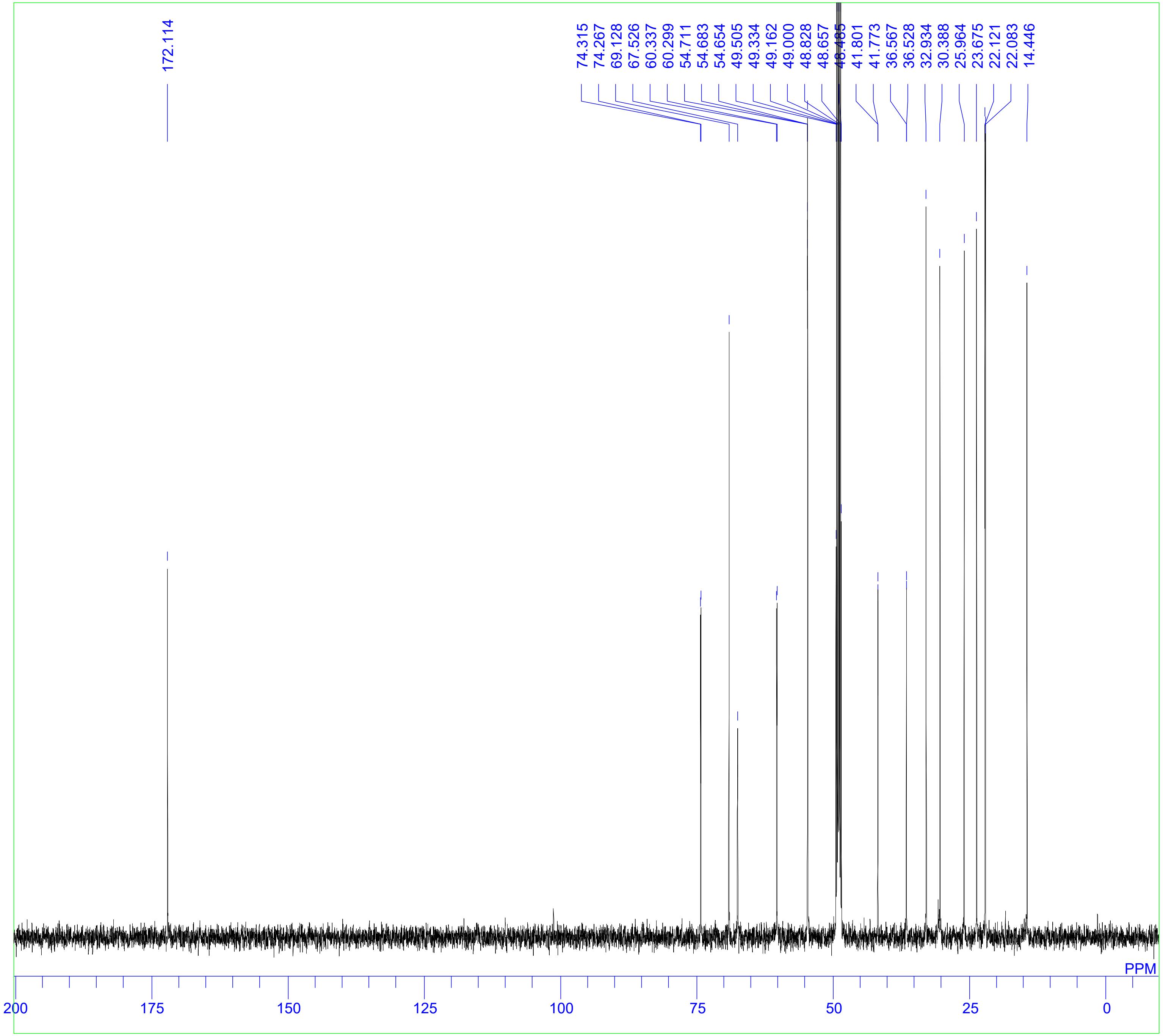

DFILE	ts-1-036-TM_Carbon-2-4.als
COMNT	single pulse decoupled gated NOE
DATIM	2015-09-14 13:39:01
OBNUC	13C
EXMOD	carbon.jxp
OBFRQ	125.77 MHz
OBSET	7.87 KHz
OBFIN	4.21 Hz
POINT	32780
FREQU	39308.18 Hz
SCANS	363
ACQTM	0.8336 sec
PD	2.0000 sec
PW1	3.58 usec
IRNUC	1H
CTEMP	24.3 c
SLVNT	CD3OD
EXREF	49.00 ppm
BF	0.12 Hz
RGAIN	22

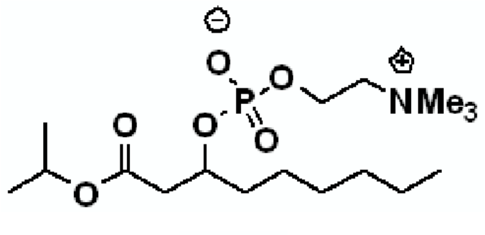

L_{deriv}
